# An experimentally representative in-silico protocol for dynamical studies of lyophilised and weakly hydrated amorphous proteins

**DOI:** 10.1038/s42004-024-01167-6

**Published:** 2024-04-12

**Authors:** Elisa Bassotti, Sara Gabrielli, Gaio Paradossi, Ester Chiessi, Mark Telling

**Affiliations:** 1 Department of Chemical Science and Technologies, University of Rome Tor Vergata, Via della Ricerca Scientifica I, 00133 Rome, Italy; 2 STFC, ISIS Facility, Rutherford Appleton Laboratory, Harwell Campus, Didcot, OX11OQX UK; 3 Department of Materials, University of Oxford, Parks Road, Oxford, UK

**Keywords:** Molecular modelling, Proteins, Computational chemistry, Bioanalytical chemistry

## Abstract

Characterization of biopolymers in both dry and weakly hydrated amorphous states has implications for the pharmaceutical industry since it provides understanding of the effect of lyophilisation on stability and biological activity. Atomistic Molecular Dynamics (MD) simulations probe structural and dynamical features related to system functionality. However, while simulations in homogenous aqueous environments are routine, dehydrated model assemblies are a challenge with systems investigated in-silico needing careful consideration; simulated systems potentially differing markedly despite seemingly negligible changes in procedure. Here we propose an in-silico protocol to model proteins in lyophilised and weakly hydrated amorphous states that is both more experimentally representative and routinely applicable. Since the outputs from MD align directly with those accessed by neutron scattering, the efficacy of the simulation protocol proposed is shown by validating against experimental neutron data for apoferritin and insulin. This work also highlights that without cooperative experimental and simulative data, development of simulative procedures using MD alone would prove most challenging.

## Abstract (web summary)

Understanding the stability and activity of freeze-dried bio-macromolecules at low degrees of hydration is crucial for pharmaceutical and food industries, however, the building of in silico models for dynamical studies at a molecular level needs careful consideration. Here, the authors propose a modelling protocol that mimics experimental protein lyophilization, and proteins in weakly hydrated amorphous states, and validate it against experimental neutron scattering data.

## Introduction

The characterisation of biopolymers in the dry amorphous state has implications for the pharmaceutical and food industries since it provides deeper understanding regarding the effect of lyophilisation on the stability and biological activity of bio-macromolecules. Indeed, while not a direct consequence of this work per se, deeper understanding of the lyophilisation process is also necessary to optimise the variables that influence the cost-effectiveness and environmental friendliness of the freeze-drying cycle.

Since data on the long-term stability of proteins and nucleic acids in lyophilised samples is limited, successful development of in-silico systems that provide information about the effect of lyophilisation on the stability and biological activity of bio-macromolecular drugs is paramount^1,2^. However, while atomistic molecular dynamics (MD) simulations in homogenous aqueous environments are routine, dehydrated model assemblies are still a challenge. There is also limited direct experimental information regarding dehydrated protein-protein assemblies to help guide the design of the system to be studied.

Pioneering and seminal works on the simulation of protein systems at low degrees of hydration date back three decades^3–11^. However, investigations reporting on simulation of the same bio-molecular system in both dry and weakly hydrated conditions are rare. To date, in simulative methodologies focussed on the characterisation of proteins’ dynamics, both dry and hydrated models are typically obtained by adding an appropriate number of water molecules to the same starting structure^12–17^ with a subsequent equilibration procedure; herein referred to as Protocol 1.

In this work, however, we propose an alternative protocol leading to a more representative comparative description of the weakly hydrated and lyophilised states. In our approach the model of the weakly hydrated system is obtained by a mild equilibration phase leading to a relaxed molecular packing, and the model dry system is built directly from the hydrated model assembly by mimicking in-silico the lyophilisation; herein referred to as Protocol 2.

To test the efficacy of the two protocols, we generated both lyophilised and weakly hydrated models of the iron storage protein, apoferritin, from 10 to 290 K. Defining the level of hydration as *h* (= gram of D_2_O per gram of protein), we define a lyophilised system as having *h* ≤ 0.05 and a weakly hydrated system as 0.05 < *h* < 0.38.

**Fig. 1:**
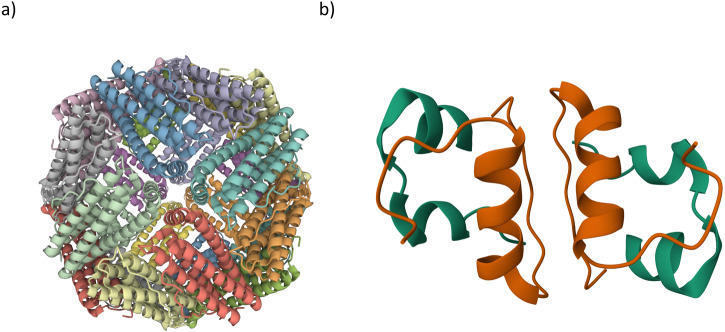
Biological assemblies of the simulated proteins. **a** Biological assembly of horse spleen apoferritin as generated using the PDB structure 2W0O. Each of the 24 subunits is represented in a different colour. The result is a 24-peptide chain (24-mer), hollow, quaternary structure of internal diameter ~75 Å. **b** PDB structure 1APH of pancreatic bovine insulin. The biological assembly corresponds to the dimeric form of insulin. Chain A of each monomer is coloured in green, while Chain B is coloured in orange.

Apoferritin, illustrated in Fig. 1a, was chosen for the complexity of its native biological assembly, its stability, hydrophobicity and its secondary structure content. Horse spleen apoferritin (MW ≈ 444,000 Da) is composed of 24 identical polypeptide chains, each having a molecular weight of 18,500 Da^18^.

Indeed, the size of the apoferritin molecule presented a simulation challenge that our team aimed to address; the peculiar shape of apoferritin offered the opportunity to study, for the lyophilised system, a native protein-vacuum internal interface, missing in many globular proteins in the dry amorphous state. Its stable, yet spherical structure, was also welcome such that it allowed radial distribution analysis to be performed in order to correlate hydration water and protein dynamics.

To authenticate experimentally the two in-silico lyophilisation protocols, and thus validate (at least dynamically) the resulting model assemblies, the temperature dependence of the mean squared displacement of the protein hydrogen atoms was chosen as the dynamical observable; this parameter being accessible via both experimental Quasi-elastic neutron scattering (QENS)^19^ measurements and simulated neutron scattering spectra extracted from the MD trajectories.

To further verify the efficiency of Protocol 2 we built lyophilised and weakly hydrated models of pancreatic bovine insulin, a low molecular weight (5800 Da for the monomer form^20^) globular protein (Fig. 1b). Human insulin, highly homologous to the bovine protein, is relevant in biopharmaceutical field for its biological function and often stored in the lyophilised state. As compared to apoferritin, insulin has a less homogeneous secondary structure, with both α-helix and β-sheet regions^21^.

Indeed, the results obtained for insulin, a small protein with a more heterogeneous secondary structure and total charge per subunit of −2^22^ (total charge of apoferritin = −7^23^), confirm the efficacy of the optimised simulation protocol.

Experimental apoferritin and insulin datasets were collected using the OSIRIS^24^ and IRIS^25^ backscattering spectrometers, at the ISIS pulsed neutron and muon source, UK, respectively. These instruments allow access to an experimental upper temporal observation limit of ~150 ps and a real space effective spatial range of ~3.5–15.2 Å.

Suitable agreement reached between experiment and simulation would lead to key dynamical changes observed experimentally, such as transition temperatures and inflection points, being better understood at a molecular level using atomistic MD analysis tools.

It is also worth noting that the hydration levels of the systems investigated in this work are sub-monolayer, i.e. *h* < 0.38^26^. While a monolayer is typically characterised by the presence of 2–3 water molecules per residue^27^, the hydrated models of apoferritin and insulin studied here have 1.7 and 1.4 water molecules per residue, respectively. Despite 2–3 water molecules per residue being the amount of water typically needed for complete recovery of biological activity, the hydration threshold for activity is commonly defined to be *h* = 0.2^28^.

Based on our findings, the approach of Protocol 2, which is both experimentally representative and routinely applicable, was successful in depicting the local dynamical behaviour of weakly hydrated and lyophilised systems. This work highlights that without cooperative experimental and simulative data, development of simulative procedures using MD alone would prove most challenging. Indeed, since direct data on the molecular re-organisation of amorphous proteins at low hydration is often unavailable, this in-silico strategy has the potential as a tool to explore features of still uncharacterised assemblies of biological macromolecules, as well as of water soluble synthetic polymer systems, in post-lyophilisation states. The work also highlights the predictive benefits of using MD simulations when evaluating past, and considering future, experiments, testing hypotheses and planning future work.

This paper is divided into three key sections (including this Introduction). The ‘Results and Discussion’ section presents and compares to experimental QENS data, dynamical information as predicted by the simulations. Guided by agreement with the neutron results, the influence of water molecules on protein dynamics at an atomistic level is then explored. Future perspectives regarding this work, are also reported. The ‘Methods’ section contains key information on molecular dynamics protocol, in terms of both construction of the models and analyses of the trajectories, and a description of the modelled proteins, respectively. This section also reports on the neutron method.

## Results and discussion

### Protocol validation: the mean squared displacement parameter

**Fig. 2:**
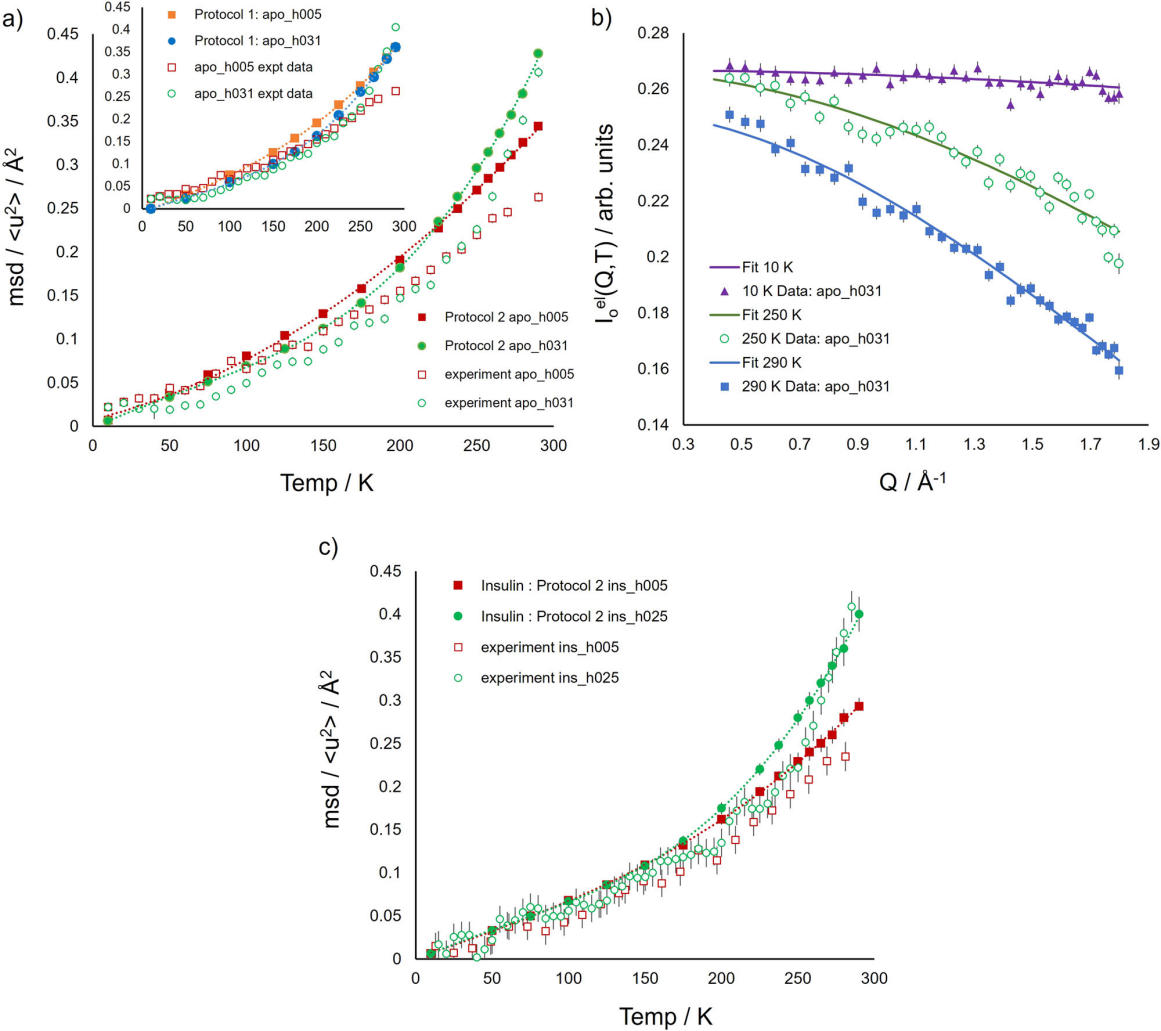
Protocol validation via comparison of the simulated and experimental mean squared displacement parameter of apoferritin and insulin. **a** Mean squared displacement parameters extracted from simulated apoferritin (MSD(T), full symbols) and experimental (<u^2^(T)>, empty symbols) neutron scattering data. apo_h005 (red squares) and apo_h031 (green circles) denote lyophilised and hydrated apoferritin, respectively. The simulated MSD(T) values presented here were extracted from MD data generated from apoferritin models constructed using Protocol 2. Inset: simulated MSD(T) values predicted from model assemblies built using Protocol 1 for apo_h005 (full orange squares) and apo_h031 (full blue circles). Dotted lines are a guide to the eye. **b** Representative experimental data for hydrated apoferritin (0.31 g of D_2_O per g of protein) and associated fits at 10 (full purple triangles), 250 (empty green circles) and 290 (full blue squares) K. Data fitted using Eq. (2) (see Methods) from which the experimental <u^2^(T)> values shown in a) were extracted. **c** Protocol 2 validation via comparison of the simulated (full symbols) and experimental (empty symbols) mean squared displacement parameter of bovine insulin. ins_h005 (red squares) and ins_h025 (green circles) denote lyophilised and hydrated insulin, respectively. The simulated MSD(T) values were extracted from MD data generated from insulin models constructed using Protocol 2. MSD(T) values and errors are averages and standard deviations over 12 independent simulation replicas. Dotted lines are a guide to the eye. Errors, when not visible, are within symbol size.

Experimental (<u^2^(T)>) and simulated (MSD(T)) mean squared displacement parameters from weakly hydrated and lyophilised apoferritin are reported as a function of temperature in Fig. 2a. It should be noted that to replicate the experimental conditions, the models of hydrated and lyophilised apoferritin, hereafter named as apo_h031 and apo_h005, respectively, contain a number of water molecules corresponding to *h* = 0.31 and 0.05, respectively, *h* being the mass of D_2_O per unit mass of protein. As mentioned in the Introduction, and described in the Methods, two simulation protocols were used to build the lyophilised and weakly hydrated protein assemblies. The MD protocols were validated by comparing mean squared displacement parameters extracted from simulated (MSD(T)) and experimental (<u^2^(T)>) neutron scattering data. Analysis shows that the difference in the temperature behaviour between hydrated and lyophilised protein, experimentally detected, is better reproduced with Protocol 2.

While the simulated MSD(T) values predicted using Protocol 2 are marginally higher than those observed experimentally, said methodology does, importantly, better correlate the structures when building the lyophilised system from the weakly hydrated model and, importantly, comparatively accentuate the dynamical transition at ~220 K^29–31^. In contrast, and as illustrated by the inset in Fig. 2a, the use of Protocol 1 suggests little difference in mobility between the lyophilised and the hydrated systems, and the water-induced dynamics enhancement experimentally detected for the hydrated system at the higher temperatures is absent. The direct comparison between the two protocols findings (Supplementary Fig. 8 in Supplementary Note 1) shows that Protocol 2 is more effective in reproducing the mean squared displacement values of lyophilised and weakly hydrated apoferritin at temperatures above 250 K. Interestingly, simulated (both protocols) and experimental results exhibit hindered mobility (i.e. MSD(T) hydrated < MSD(T) lyophilised) in the hydrated state at *T* < 175 K. However, with Protocol 1 the mobility attenuation of the hydrated system extends over all the temperature range, in contrast to the experimental behaviour.

To further test the efficacy of Protocol 2, we simulated bovine insulin in the lyophilised (*h* = 0.05) and weakly hydrated (*h* = 0.25) states. As compared to apoferritin, bovine insulin is markedly different and has a composite secondary structure, with both α-helix, 3_10_-helix and β-sheet regions^21^. In the simulation model we described the biological assembly as a dimer, considering the insulin aggregation propensity in zinc-free solution^20^. Experimental (<u^2^(T)>) and simulated (MSD(T)) mean squared displacement parameters from hydrated and lyophilised insulin are compared as a function of temperature in Fig. 2c. Simulation results are in good agreement with experimental findings, detecting an enhancement of mobility of hydrated insulin model, ins_h025, as compared to the lyophilised insulin model, ins_h005, at temperatures above 225 K.

**Fig. 3:**
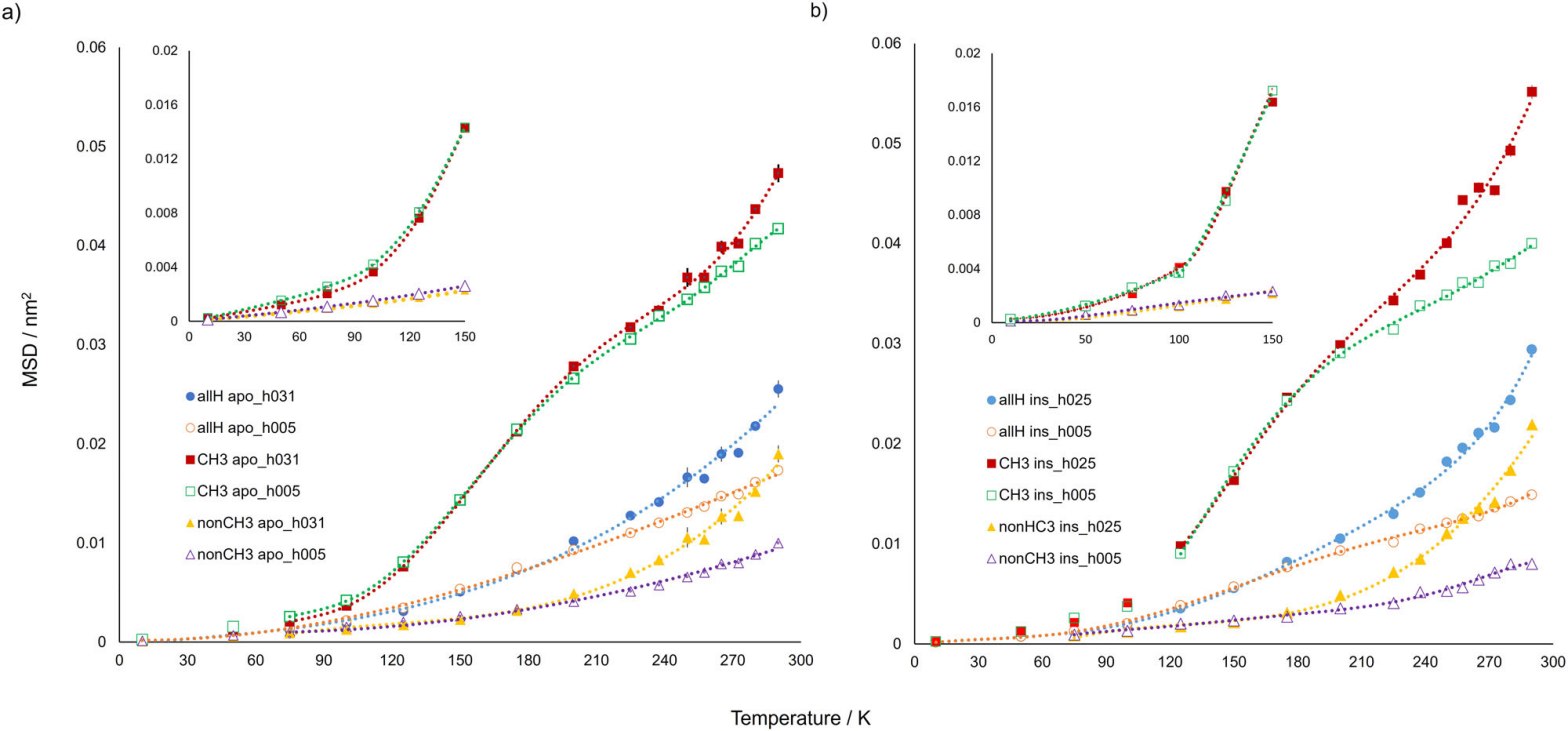
MSD_t_(T) for all hydrogen atoms, methyl hydrogens and non-methyl hydrogens over 6–8 ns. **a** MSD_t_(T) for all hydrogen atoms (all-H apo_h031, full blue circles, and all_H apo_h005, empty orange circles), methyl hydrogens (CH_3_ apo_h031, full red squares, and CH_3_ apo_h005, empty green squares) and non-methyl hydrogens (non-CH_3_ apo_h031, full yellow triangles, and non-CH_3_ apo_h005, empty purple triangles) over 6-8 ns for apoferritin. Inset: MSD_t_(T) for CH_3_ and non-CH_3_ in the temperature range from 10 to 150 K. Errors are obtained using the blocking method and the lines are a guide to the eye. **b** MSD_t_(T) for all hydrogen atoms (all-H ins_h025, full blue circles, and all-H ins_h005, empty orange circles), methyl hydrogens (CH_3_ ins_h025, full red squares, and CH_3_ ins_h005, empty green squares) and non-methyl hydrogens (non-CH_3_ ins_h025, full yellow triangles, and non-CH_3_ ins_h005, empty purple triangles) over 6–8 ns for insulin. Inset: MSD_t_(T) for CH_3_ and non-CH_3_ in the temperature range from 10 to 150 K. Values and errors are averages and standard errors, respectively, over 12 independent simulation replicas. The lines are a guide to the eye. Errors, when not visible, are within symbol size.

It should be mentioned that the presence of a sub-monolayer water content determines the enhancement of apoferritin and insulin mobility, visible above the dynamical transition temperature both in the experimental and simulation-derived mean square displacement of protein hydrogen atoms. The plasticizing effect of water is detected also in the direct trajectory analysis on a temporal range wider than that explored by the experiment (Fig. 3), as expected^27^. The extent of the mobility increase of apoferritin at *h* = 0.31 as compared to the dry state, estimated from the ratio of the <u^2^(T)> values at 290 K (Fig. 2a) is about 1.6, similar to that seen for insulin at *h* = 0.25 (Fig. 2c). An analogous relative increment of mobility upon hydration has been detected for lysozyme at *h* = 0.40 and 300 K by elastic neutron scattering experiments using an energy resolution^32^ of the same order of magnitude of that of our study. This suggests that, in the picosecond time scale explored in these experiments, the magnitude of the plasticizing effect of water does not depend on the complexity of the bio-molecular assembly.

### Protocol validation: force field comparison

The OPLS-AA force field was chosen for this work due to its suitability to also describe non-biological systems^33^ thus making it possible to extend our Protocol 2 to synthetic macromolecules. However, any possible influence of force field choice on the simulation of hydrogen dynamics is an important consideration. We therefore repeated the Protocol 2 simulations using the CHARMM v27 all-atom force field^34^ and the TIP3P water model^35^; the same force field setup as used for the simulations reported by ref. ^16^. Mean squared displacement (MSD) parameters extracted from simulated neutron scattering data for apo_h031 and apo_h005 using the OPLS-AA^36^ and CHARMM v27 force fields are compared in Supplementary Fig. 9 (Supplementary Note 2). We find that the choice of force field only weakly affects apoferritin hydrogen displacement amplitudes, those obtained with CHARMM v27 being marginally lower. We believe this small discrepancy can be ascribed to differences in the functions describing the potential energy components and in the parameters, such as partial charges, between these force fields. Nonetheless, the experiment-simulation agreement obtained with OPLS-AA and CHARMM v27 is similar.

Given the better overall agreement between experiment and simulation using Protocol 2, the origin of transition temperatures and dynamical discontinuities observed in the experimental data were further investigated at a molecular level using atomistic MD analysis tools.

### Dynamical properties from direct space trajectory analysis (6–8 ns)

Direct (time domain) analysis of all the MD trajectories generated using Protocol 2 was performed in the time interval 6–8 ns (see Supplementary Methods 2.3). This work was to further understand, at a molecular level, the mechanisms driving the transition temperatures and inflexion points observed in the temperature behaviour of the mean squared displacement (msd) extracted from simulated (MSD(T)) and experimental (<u^2^(T)>) neutron scattering data.

From here on the mean msd values obtained from direct space (aka time domain) analysis are named MSD_t_(T) to distinguish them from the MSD(T) and <u^2^(T)> msd parameters obtained from the simulated and experimental neutron data. Average MSD_t_(*T*) values were calculated from the trajectory files by monitoring the time evolution of the mean squared displacement parameter, MSD(t). The method of extracting MSD_t_(T) from MSD(t) data is described in detail in the Supplementary Information (see Supplementary Methods 2.3).

The temperature evolution of MSD_t_(*T*) was evaluated not only for all-hydrogen atoms but also for just those H atoms associated with i) methyl and ii) non-methyl species. MSD_t_(T) was determined for said subsets to ascertain, as a function of temperature, their respective and relative contributions to the global (i.e. all-H atom) protein dynamics.

Considering all hydrogen atom analysis first, Fig. 3a shows that MSD_t_(T) for both lyophilised and weakly hydrated apoferritin follows an almost linear response from 10 to 100 K. Deviation from linearity is observed at *T* ~ 100 K, which is indicative of methyl group activation, as later confirmed by selective analysis of methyl and non-methyl hydrogen msds. Above 100 K, the evolution of MSD_t_(T) is accentuated for apo_h031, an exponential-like increase in magnitude as a function of temperature being observed. In contrast, MSD_t_(T) for apo_h005 appears linear-like up to 290 K. A second dynamical discontinuity is evident above 100 K; MSD_t_(T) for apo_h031 deviating from that of apo_h005 at ~225 K. This observation agrees with previous work on weakly hydrated biomaterials, with the gradient increase at 225 K being indicative of thermal activation corresponding to the so-called dynamical transition^13,29,31,37–41^.

Further inspection of Fig. 3a corroborates MSD(T) and <u^2^(T)> observation (in Fig. 2) that hydration appears to have two opposing effects. While there is a clear mobility enhancement upon hydration at temperatures greater than ~220 K, protein motion in apo_h031 is weakly hindered by the presence of water molecules at low temperatures (*T* < 175 K). Here the lyophilised protein is seen to be slightly more mobile than the hydrated system (see Fig. 3a (inset)), a finding confirmed by the residue-resolved analysis of mobility at 150 K, as later reported.

To better understand which species are responsible for the dynamical changes seen for the whole ensemble of hydrogen atoms, mean squared displacement analysis was repeated to consider first MSD_t_(T) of hydrogen atoms from only CH_3_ groups and then from the other non-methyl associated functional groups. Obviously, the mean squared response from all hydrogen atoms corresponds to the weighted average of these two methyl/non-methyl components according to their relative population. Indeed, Fig. 3a shows that the mean squared displacement associated with methyl group hydrogen atoms is greater than that of those hydrogen atoms associated with other functional groups. Such a result might be explained by conjecting that the internal rotation of methyl groups couples to the segmental motion of the biopolymer; such amplification missing for those hydrogen atoms associated with non-CH_3_ moieties. The behaviour of MSD_t_(T) for methyl hydrogens displays a single transition at 100 K for both apo_h031 and apo_h005. This result confirms that the discontinuity observed at 100 K is associated with the activation of methyl dynamics as observed for proteins with different secondary structure compositions^42^. It is noteworthy that the MSD_t_(T) of non-methylated species lacks such transition.

Considering now just the behaviour of MSD_t_(T) from non-methyl hydrogens in apo_h031 and apo_h005, a dynamical discontinuity is observed at about 225 K only in the weakly hydrated state, which aligns with the response detected for MSD_t_(T) for all hydrogen atoms. This result supports the common interpretation of the dynamical transition^29,43,44^ namely that it is a kinetically controlled process where segmental and collective motions involving, in particular, non-methyl hydrogens start to evolve.

Concerning the inversion observed in MSD_t_(T) between 75 and 175 K, where the mobility of lyophilised system is slightly greater, separation of methyl and non-methyl contributions shows that this response is most likely ascribed to the attenuation of methyl group motion in apo_h031; the MSD_t_(T) values of non-methyl hydrogens in apo_h031 and apo_h005 in this temperature range being almost coincident (Fig. 3a (inset)). Interestingly, and as discussed in the following section, the mechanism by which water acts on methyl motions must be mediated by the hydration of other protein moieties since methyl groups are not directly exposed to water.

The same analysis of the MSD_t_(T) dynamical observable was performed for the insulin model systems. The results are qualitatively similar to those of apoferritin (Fig. 3b) but the overall mobility of weakly hydrated insulin above the dynamical transition is slightly larger as compared to hydrated apoferritin, the opposite holding for the dry states. In particular, two features distinguish the dynamical behaviour of these proteins:

i. No water-induced mobility reduction is detected for insulin at low temperature (inset of Fig. 3b), in agreement to what experimentally observed within error (Fig. 2c)
ii. In the hydrated system, the dynamical transition causes a significant mobility enhancement of not only non-methyl hydrogen atoms but also of methyl hydrogen atoms, since above 225 K the MSD_t_(T) of this class of hydrogen atoms is much higher in ins_h025 as compared to ins_h005.

As discussed below, finding (ii) is confirmed by the subsequent analysis of MSD of methyl hydrogen atoms at the time of 1 ns, where it is evident that the mobility increase of insulin methyl hydrogen atoms at the dynamical transition, undetected for apoferritin, is determined by non-rotational motions of methyl groups.

### Direct space trajectory analysis at 1 nanosecond: water-induced hindrance at 150 K

Concerning the water-induced hindering effect of water at low temperature observed in apoferritin, the reduction of small amplitude, fast motions of biomolecules at low temperature, as compared to the dry (lyophilised) state, has been experimentally observed at temperatures lower than 170 K for other weakly hydrated proteins, such as lysozyme^13,45^, RNase A^46^, myoglobin^47,48^, pig liver esterase^49^ and Green Fluorescent Protein (GFP)^50^. Such findings indicate the apparent dynamical anomaly we observed for apoferritin is a relatively general phenomenon in dry amorphous proteins. More importantly, however, it is detected in systems that have been lyophilised from D_2_O solution*;* our proteins, in contrast, being lyophilised from H_2_O solution and then weakly hydrated using D_2_O (see Supplementary Methods 4: The Apoferritin and Insulin Molecules: Neutron Experiment Sample Preparation).

It should be noted that one needs to be mindful regarding the possible influence of exchangeable moieties in systems lyophilised from H_2_O solution but weakly hydrated using D_2_O. However, in our case we show from trajectory manipulation (Supplementary Note 8: Simulated Effect of H/D Exchange in Weakly Hydrated Apoferritin) that, at *h* = 0.31, such influence is minimal. The ability to perform such calculations highlights the predictive benefits of using MD simulations when evaluating past, and considering future, experiments, testing hypotheses and planning future work.

It is noteworthy that a considerable effect of mobility attenuation at 150 K was observed for GFP at *h* = 0.40^50^, whose magnitude is similar to that we detect for apoferritin at *h* = 0.31 (Fig. 2a) in the same temperature region. The barrel-like structure of GFP, composed of β-sheet regions, has been hypothesised to be a possible reason for the much-pronounced suppression of the MSD at low temperatures detected for this protein. Our results, however, show that while apoferritin exhibits at low-temperature water-induced mobility decrease comparable to that of GFP, it does so in a system practically devoid of β-sheet regions and rich in α-helices. This suggests that other characteristics of the protein can determine the extent of this phenomenon, such as the topology of the bio macromolecule surface. In this respect, no mobility attenuation at low temperature can be detected for hydrated insulin (Fig. 3b) neither from the behaviour of simulated MSD(T) nor within the precision of the experimental data.

**Fig. 4:**
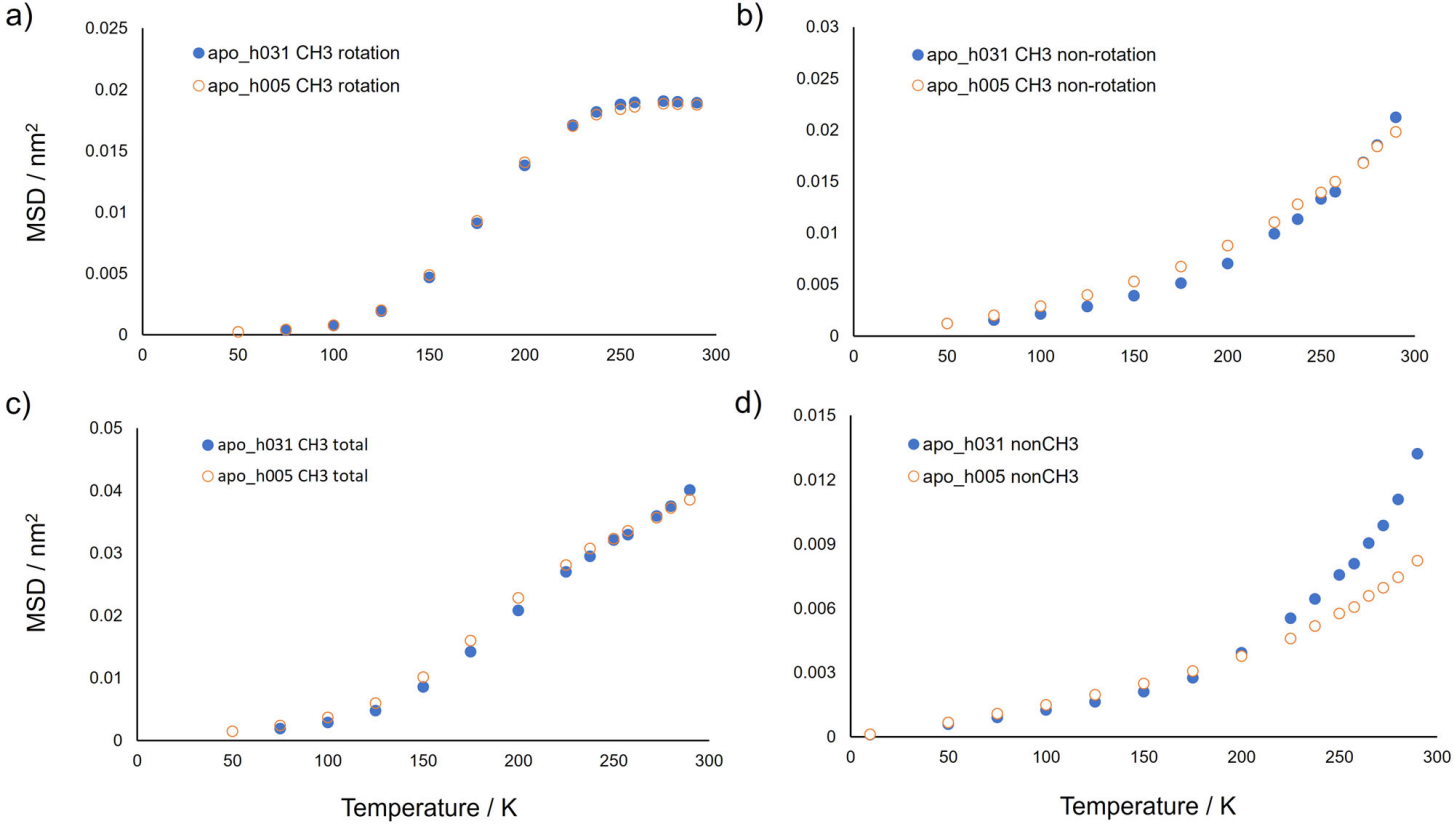
Mean squared displacements of methyl and non-methyl hydrogens for apoferritin at *t* = 1 ns. Mean squared displacements of methyl (CH_3_) and non-methyl (nonCH_3_) hydrogens for apo_h031 (full blue circles) and apo_h005 (empty orange circles) at *t* = 1 ns. **a** MSD for rotational motions of methyl hydrogens. **b** MSD for non-rotational motions of methyl hydrogens. **c** Total MSD of methyl hydrogens. **d** MSD for non-methyl hydrogens. Errors are within symbol size.

As described above, the analysis of the mean squared displacement of methyl hydrogen atoms computed directly from the simulation trajectory in the 6-8 ns temporal interval shows that, for apoferritin, their mobility is attenuated at low temperature in the presence of hydration water (Fig. 3a). The mean squared displacement of methyl hydrogen atoms deriving from only the rotation motion of methyl groups has been computed from simulations of GFP in the dry and weakly hydrated (*h* = 0.4) states^16^. In the time widow of 1 ns no water-induced difference was found for the rotational mean squared displacement of GFP methyl hydrogen atoms. We therefore conducted a similar analysis as in ref. ^16^, by separately determining the contributions of rotation and non-rotation motions for the mean squared displacement of methyl hydrogen atoms in apoferritin at 1 ns. The results, reported in Fig. 4(a-d), show that the low-temperature mobility hindrance of water does not involve the rotation motion of methyl groups since the rotational MSDs of lyophilised and weakly hydrated apoferritin are comparable over the whole temperature interval. The same finding has been detected for GFP^16^. However, the water-induced hindrance strongly affects the non-rotational motion of apoferritin methyl hydrogen atoms, i.e. the intrinsic mobility of the carbon atom of methyl group, being this MSD component lower in apo_h031 than in apo_h005 at temperatures from 100 to 225 K (Fig. 4b).

Moreover, the rotation-deprived MSD of methyl hydrogen atoms (Fig. 4b) can be compared to the MSD values of non-methyl hydrogen atoms, intrinsically not affected by rotational motions (Fig. 4d). The comparison highlights a higher intrinsic mobility of the carbon atom of methyl group, with respect to the mobility of other chemical groups, for both apo_h031 and apo_h005 in the whole temperature range. This finding can be explained by the greater conformational freedom of side-chain ends, where methyl groups are located, as compared to the segmental mobility of more internal portions of residues and of polypeptide backbone. Finally, Fig. 4d shows a small reduction of mobility also for non-methyl hydrogen atoms of apo_h031 as compared to apo_h005 in the temperature interval 100–175 K. Such water-induced effect, much lower than that detected for methyl hydrogens, is not visible for non-methyl hydrogen atoms at the larger time scale of 6–8 ns (Fig. 3a).

**Fig. 5:**
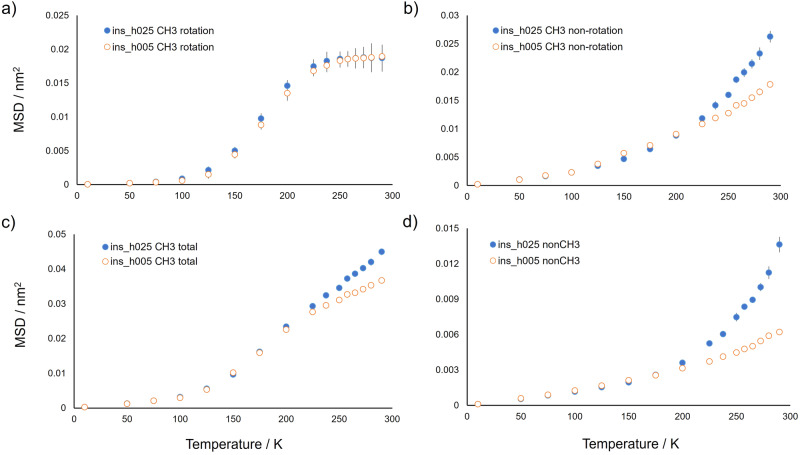
Mean squared displacements of methyl and non-methyl hydrogens for insulin at *t* = 1 ns. Mean squared displacements of methyl and non-methyl hydrogens for ins_h025 (full blue circles) and ins_h005 (empty orange circles) at *t* = 1 ns. **a** MSD for rotational motions of methyl hydrogens. **b** MSD for non-rotational motions of methyl hydrogens. **c** Total MSD of methyl hydrogens. **d** MSD for non-methyl hydrogens. Values and errors are averages and standard errors, respectively, from 12 independent simulation replicas. Errors, when not visible, are within symbol size.

The percentage of methyl hydrogen atoms over the total content of protein hydrogen atoms is very similar in apoferritin and insulin, i.e. 23% and 22%, respectively. We, therefore, analysed the MSD components of methyl hydrogen atoms also for lyophilised and weakly hydrated insulin models, ins_h005 and ins_h025, respectively (Fig. 5a–d). The temperature behaviour of the rotational component of MSD for insulin methyl hydrogen atoms is analogous to that observed for apoferritin. In particular, the upper limit value of the rotation MSD, reached for both proteins at temperatures above 250 K, is consistent with the free rotation of all methyl groups. A difference between apoferritin and insulin emerges in the non-rotational component of methyl hydrogen atoms. By considering the response of the hydrated protein to the dynamical transition, occurring for both proteins at about 220 K, the non-rotational MSD component of ins_h025 methyl hydrogen atoms has a pronounced increase and diverges from that of ins_h005 (Fig. 5b) at temperatures above the dynamical transition temperature. This mobility enhancement, as compared to the lyophilised system, is not detected for the non-rotational motions of methyl hydrogen atoms of apo_h031, the non-rotational MSD values being similar in apo_h005 and apo_h031 above 250 K. We can conclude that, for apoferritin, the onset of anharmonic motions at the dynamical transition involves mainly non-methyl hydrogen atoms, and their chemical moieties, whilst for insulin also the non-rotational mobility of methyl hydrogen atoms undergoes an increase. Such difference could be ascribed to a different exposition to water of methyl groups between these proteins in their weakly hydrated state. However, methyl-containing amino acids of ins_h025 are less hydrated as compared to other residues (Supplementary Fig. 12 in Supplementary Note 5), similarly to what found for apo_h031 (Fig. 7 and Supplementary Fig. 11). Therefore, the origin of the different dynamical behaviour of methyl groups at the dynamical transition is probably related to the larger subunit size and higher structural complexity of biological assembly for apoferritin, as compared to insulin, making methyl groups more rigid (see Supplementary Fig. 13 in Supplementary Note 6).

The presence of water is generally expected to have a plasticizing effect, which is contradicted by our results in the low temperature interval but confirmed above the dynamical transition temperature. In order to further clarify these aspects, we explored the distribution of water in hydrated apoferritin, distinguishing between methyl containing and not containing residues.

### Apoferritin radial distribution function (RDF) analysis

The pseudo-spherical symmetry of apoferritin (see Fig. 1a) allows structural characterisation to also be performed in terms of the distribution of atoms/components with respect to the centre of the biological assembly. As a result, Radial Distribution Functions (RDFs), which consider the radial distribution of specific species in the apo_h031 and apo_h005 assemblies relative to the protein’s Center Of Mass (COM), were computed and compared to those predicted using the crystallographic structure. Details of the Radial Distribution Function analysis procedure are given in the Supplementary Information (see Supplementary Methods 2.2).

**Fig. 6:**
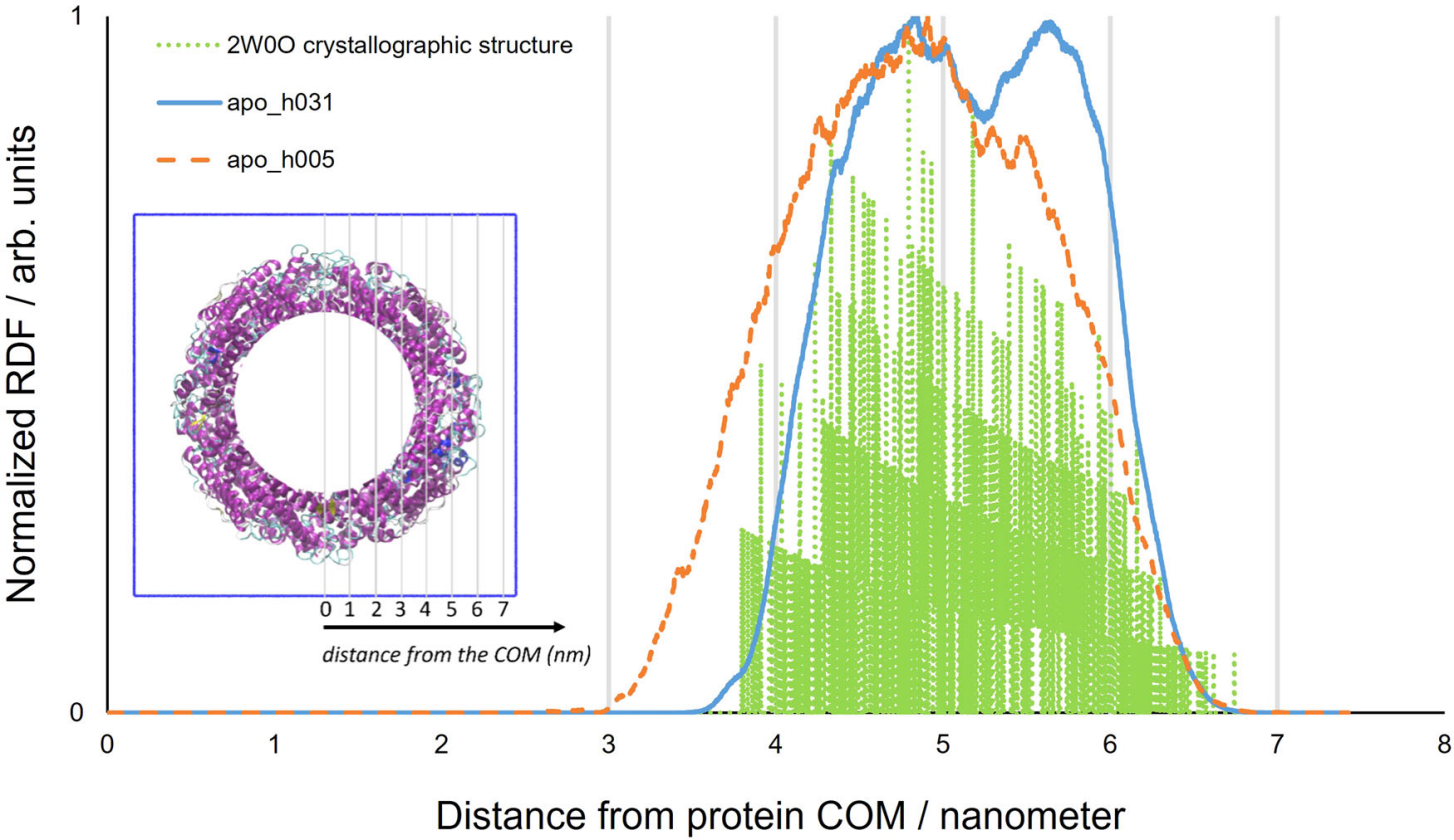
Non-hydrogen atom RDFs for apoferritin. Non-hydrogen atom RDFs for apo_h031 (solid blue line), apo_h005 (dashed orange line) and the 2W0O apoferritin crystallographic structure (dotted green line). The COM of the apoferritin assembly was chosen as the point of reference. T = 290 K. Inset: the spatial grading, relative to the COM, used in the main figure, projected onto the apo_h031 cavity.

The results obtained at 290 K, and which consider the radial distribution of all non-hydrogen (see Supplementary Methods 2.2) protein atoms (Fig. 6), show that the cavity of the hydrated model, having a radius of 3.6 nm, is similar to that of the crystallographic structure (3.8 nm). The cavity radius, however, decreases to 3 nm in the lyophilised system, further illustrating the stabilising effect of water.

### Water distribution vs. mobility

**Fig. 7:**
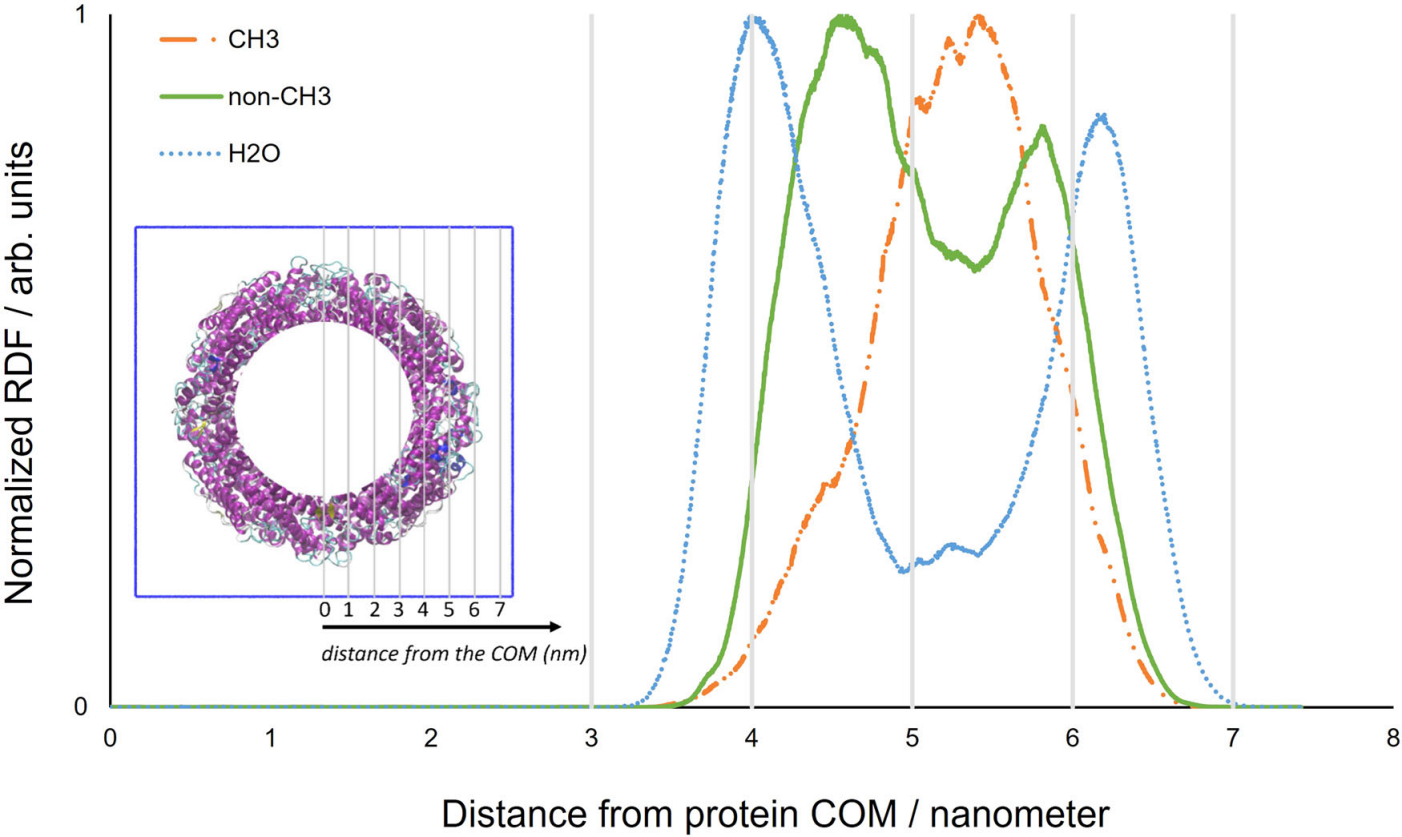
RDFs of amino acids with, and without, methyl groups in apo_h031. RDFs of amino acids with (CH_3_, dash dot orange line) and without (non-CH_3_, solid green line) methyl groups in apo_h031. The RDF of oxygen atoms of water molecules (*h* = 0.31) is also shown (dotted blue line). *T* = 290 K. The COM of the protein was chosen as the point of reference. Inset: the spatial grading, relative to the COM used in the main figure, projected onto the apo_h031 cavity.

To evaluate which, if any, of methyl and non-methyl containing residue sets might contribute to enhance protein mobility due to their proximity to water, RDF analysis was performed; the apoferritin shell position of amino acids with, and without, methyl groups being predicted. Said RDFs were computed for apo_h031 at 150 K and 290 K with the COM of the protein again being used as the point of reference. The data was compared to the radial distribution of water molecules, as shown in Fig. 7.

Considering the RDF of water-associated oxygen atoms, we find that water molecules congregate predominantly at the internal and external protein-vacuum interfaces. The distribution of non-methyl residues broadly correlates with the water molecule distribution. In contrast, we find that those amino species comprising methyl groups appear to locate in the internal region of the protein shell, which is, as seen, less accessible to water. Results comparable to those shown in Fig. 7 were also obtained for hydrated apoferritin at 150 K (see Supplementary Note 4). The fact that methyl associated residues appear to be further from water, despite methylated species giving the greater contribution to MSD_t_(T) in terms of displacement amplitude from 10 to 290 K (Fig. 3a), suggests that any hydration induced effect related to methyl species (i.e. hindrance at low temperature) is an indirect consequence of water molecule inclusion. In contrast, the positions of those residues that show the greatest enhancement of mobility at 290 K correlate with water molecules distribution, as illustrated in the following.

**Fig. 8:**
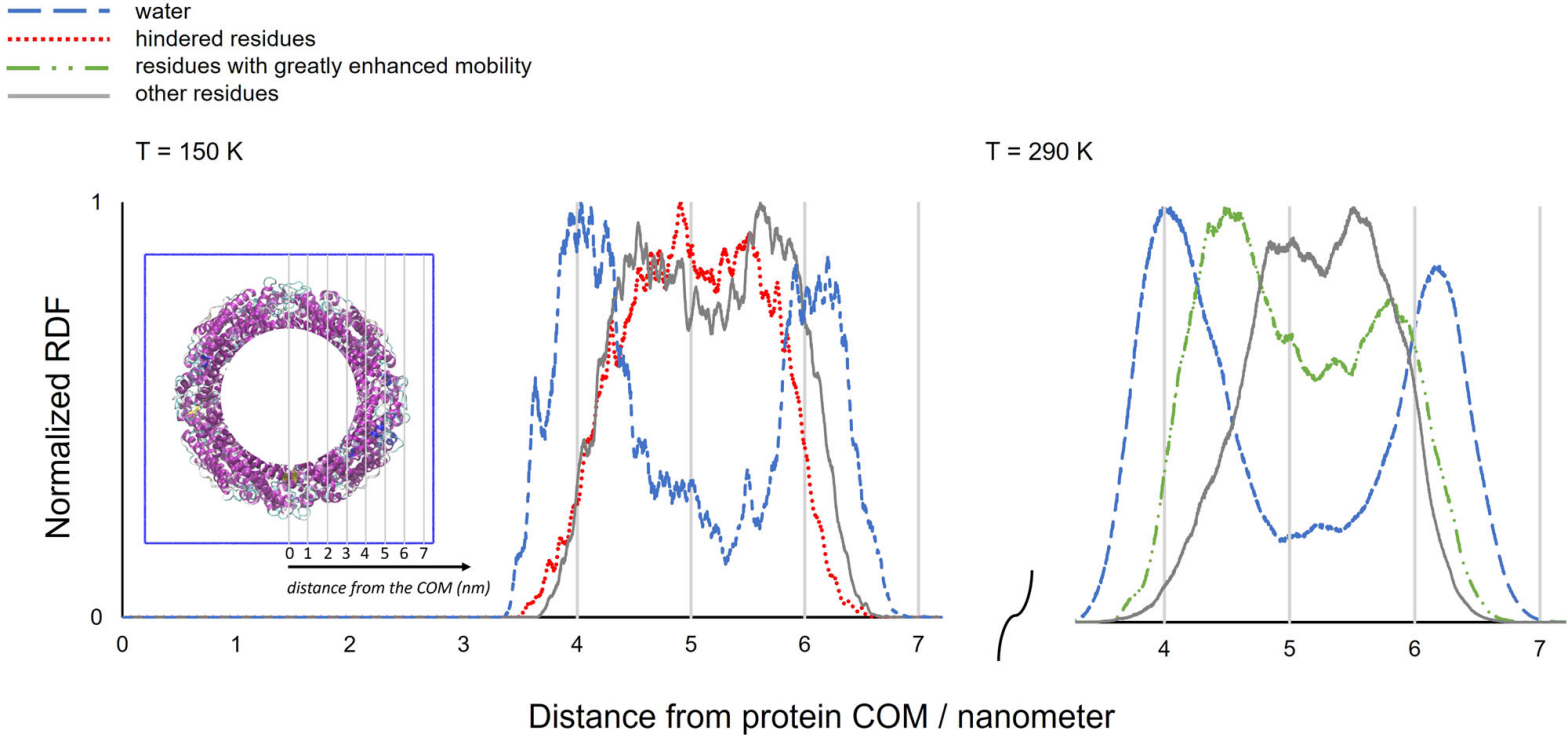
RDFs of apo_h031 for hindered residues at 150 K and for those with greatly enhanced mobility at 290 K. RDFs of apo_h031 for hindered residues at 150 K (dotted red line) and those with greatly enhanced mobility at 290 K (dash dot green line). The data sets are compared to the RDFs of water (dashed blue line) and of all other residues (solid grey line). The COM of the protein was chosen as the point of reference. Inset: the spatial grading, relative to the COM and used in the main figure, projected onto the apo_h031 cavity.

As shown experimentally in Fig. 2, the effect of hydration on protein mobility depends on temperature. At higher temperatures, solvation causes enhancement of the mean squared displacement parameter, whilst around 150 K a slight depression in <u^2^(T)> is observed; a result replicated in the simulations (Figs. 2a and 3a). These effects were also investigated using RDF analysis by characterising each individual apoferritin protein residue in terms of variation in mobility upon hydration, relative to their dry state motion at a common temperature. The change in residue mobility was defined using:

1 $${change}\,{in}\,{mobility}\left( \% \right)=\frac{{{MSD}}_{{ap}{o}_{h031}}-{{MSD}}_{{ap}{o}_{h005}}}{{{MSD}}_{{ap}{o}_{h005}}}100$$

the residue MSD being computed in the 6–8 ns time interval. RDFs of the hydrated model were calculated for those amino acids with hindered mobility at 150 K (<−10% change in mobility) and for those with greatly enhanced mobility (>+60% change in mobility) at 290 K. With these criteria, we sample the most affected residues of apo_h031 in terms of mobility attenuation or enhancement by hydration, at 150 or 290 K, respectively. Residue ensembles selected at 150 and 290 K involve 53% and 45% of apoferritin residues, respectively, showing that in both cases a considerable part of the protein is involved. The COM of the protein was chosen as the point of reference for the RDFs of the selected residue ensembles at 150 and 290 K, shown in Fig. 8.

Each RDF was also compared to that of water and to that of all other (i.e. non-influenced) residues (Fig. 8). Hindered residues at 150 K do not appear to be near water, suggesting that there is an indirect effect of hydration on mobility attenuation at low temperature. In contrast, the RDF for the most influenced residues at 290 K shows two broad peaks that correlate with water molecule distribution. This result suggests that water proximity directly impacts the mobility of these particular species.

### Mobility of hydration water

Having analysed protein mobility, we now exploit the apo_h031 and ins_h025 simulations to predict the mobility of hydration water; the results being likened to those reported for weakly hydrated cytochrome P450^51^.

**Fig. 9:**
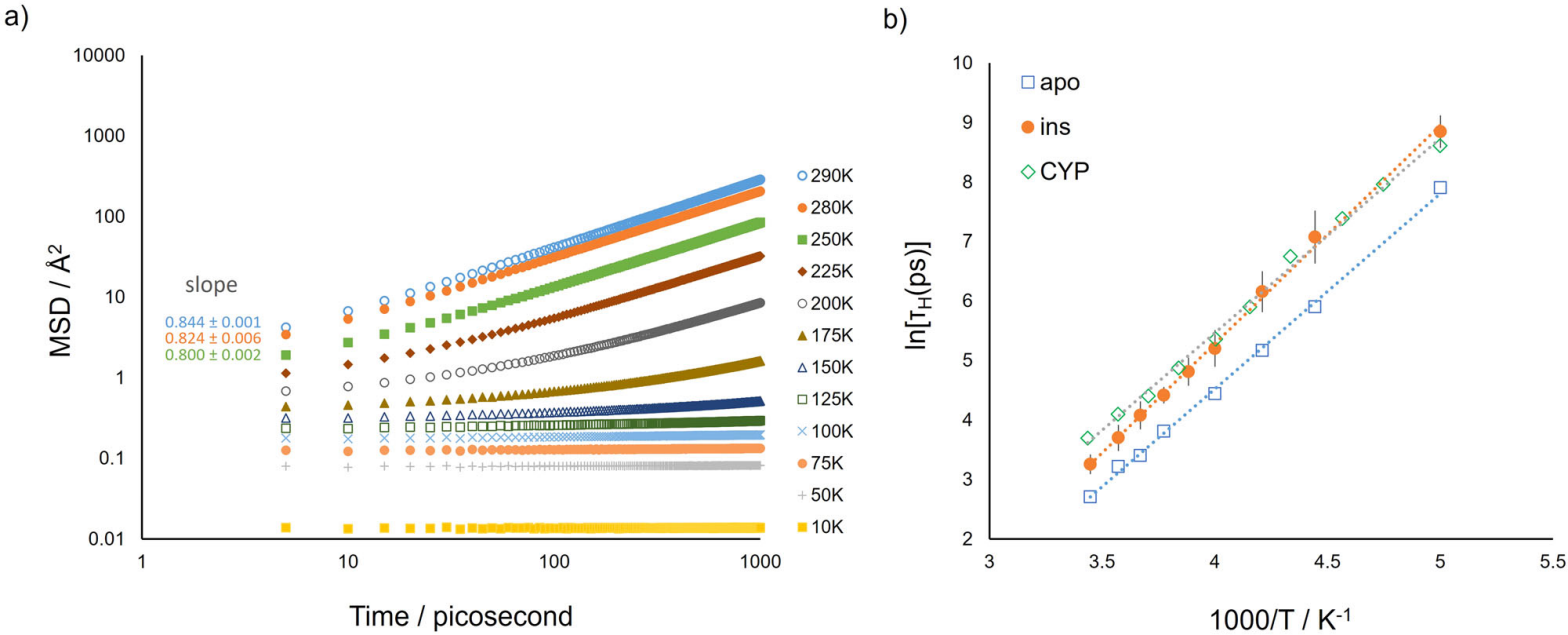
Computational observables of hydration water mobility. **a** MSD of water oxygen atoms in a time interval of 1 ns at temperatures between 10 and 290 K for the apo_h031 model. The exponent of the power law fit of the MSD vs time behaviour is reported for the higher temperatures. **b** Characteristic relaxation time, τ_H_, of water-water hydrogen bond in weakly hydrated proteins as a function of temperature. Apoferritin (apo_h031, empty blue squares), insulin (ins_h025, full orange circles) and cytochrome P450 (data from Chem. Sci., 2022, 13, 4341, CYP, empty green diamonds) values. *τ*_H_ is defined as the time when the corresponding hydrogen bond correlation function *C*_H_(t) decays to e^−1^. Values and errors for insulin are averages and standard deviations from 12 independent simulation replicas. Where not visible, error bars are within symbol size.

Because of the sub-monolayer water content of these systems, all water molecules can be considered as protein hydration water^27^. The MD simulation-derived MSD of apoferritin hydration water in the time window from 5 ps to 1 ns for temperatures from 10 to 290 K is shown in Fig. 9a. The exponent of the power law fit of the MSD vs time behaviour is lower than unity also for the highest investigated temperatures, indicating a sub-diffusive behaviour. Water molecules in the ins_h025 model show the same dynamical behaviour (see Supplementary Fig. 5). An analogous result has been reported for green fluorescent protein (GFP) and cytochrome P450 (CYP), whose hydration water is characterised by diffusion in a restricted area or volume or subdiffusion^52,53^. Therefore the sub-diffusive motion of hydration water in proteins proves itself to be a general feature irrespective of the biomacromolecule secondary and tertiary structures, which are extremely different in GFP, CYP, apoferritin and insulin. The MD simulation-derived MSD value of water at 280 K for GFP with *h* = 0.40 is slightly lower than what we find for apo_h031^52^ which could be related to a different water-protein interface, being apoferritin and GFP predominantly composed of α-helix and β-sheet regions, respectively. The results in Fig. 9a and Supplementary Fig. 5 show that water dynamics in apoferritin and insulin becomes evident at 200 K, close to the dynamical transition (*T* ~ 220 K). This observation might suggest a dynamical coupling between the protein and water^40^, similarly to the interplay of protein and molecular environment detected for lysozyme in glycerol-water glassy mixtures^54^. However, the onset temperature of water dynamics in our systems coincides with the result of a recent study of weakly hydrated materials, stating, by experimental and simulation evidence, a universal onset of water mobility irrespective of the chemical nature of the hydrated interface^51^. This correspondence is noteworthy, considering the difference of systems and of water computational model between our work and that in ref. ^51^. To further characterise the water behaviour, we computed the water–water hydrogen bond correlation function *C*_H_(t), as described in Supplementary Methods 2.4. The time decay of this function is due to the switching of inter-water hydrogen bonds, occurring with a characteristic relaxation time, *τ*_H_, defined as the time when the correlation function *C*_H_(t) reaches the value of e^−1^ (see Supplementary Fig. 6). By plotting ln(*τ*_H_) as a function of inverse temperature, and fitting the Arrhenius form, an associated water molecule activation energy, *E*_a_, of 27.3 ± 0.5 kJ mol^−1^ in the temperature range 200 to 290 K could be ascertained (Fig. 9b). The same analysis performed for the hydration water of weakly hydrated insulin provides an activation energy of 30.5 ± 0.5 kJ mol^−1^ (Fig. 9b). The values of *τ*_H_ and *E*_a_ obtained for apo_h031 and ins_h025 are consistent to that found for hydration water in simulations of weakly hydrated cytochrome P450 and graphene oxide membrane^51^, again supporting the occurrence of a surface independent relaxation process in water with an approximately universal energy barrier of 30 kJ mol^−1^. A final comment concerns a difference between apoferritin and the other proteins visible in Fig. 9b. The characteristic relaxation time of hydrogen bonding between water molecules on the surface of apoferritin is lower, showing a faster dynamics of hydration water for this protein. It is noteworthy that apoferritin was found to display a superior ice-nucleation ability at sub-zero temperatures as compared to other proteins^55^. The faster water dynamics detected by simulations suggests a weaker adsorption of water on apoferritin as compared to insulin and CYP, making easier the ice-nucleation at the protein interface.

The interplay of complementary experimental and simulative methods was paramount to the success of this project. The QENS technique is supremely sensitive to the motion of hydrogen-rich species and provides direct observation of the mobility of biopolymers in weakly hydrated environments. Indeed, better understanding at a molecular level of the key dynamical changes observed experimentally, such as transition temperatures and inflection points, are reliant on atomistic MD analysis tools. However, to generate comparative systems using MD simulations alone, without guidance, would have proved most challenging; the features to be reproduced in simulated systems can differ markedly upon seemingly negligible changes in the chemical composition or water molecule concentration of the models.

Given our findings, we suggest here an approach to simulate weakly hydrated and corresponding lyophilised protein models, which differs from the more established methodology of simply adding a suitable number of water molecules to the same initial model structure. In the formulation of Protocol 2 we have been guided by a key objective to obtain a realistic representation of the molecular packing of weakly hydrated and lyophilised systems. Direct information on this characteristic is missing for many amorphous proteins. As such, we adopted i) a mild equilibration procedure for the hydrated system and ii) an in-silico dehydration of the hydrated system which mimics an in vitro lyophilisation process. This approach leads to a more relaxed molecular packing in the hydrated model and allows for a structural evolution (during dehydration) which is more representative of that expected experimentally.

Indeed, our more correlated methodology allows us to disclose differences in the dynamics of apoferritin and insulin at a sub-monolayer level of hydration, which is not evident in the temporal window explored by experiments alone (Fig. 2a, c). As example, on a few nanosecond time interval (Figs. 3–5), weakly hydrated insulin displays a higher mobility above the dynamical transition, mainly because of the greater motion of methyl groups. It is noteworthy that about 50% of methyl containing residues of insulin are involved in the binding to the insulin receptor^56^. Therefore, the mobility difference between these proteins could be related to their different biological functionality; apoferritin being devoted to iron storage^57^, needing to maintain a stable, rigid structure, while insulin, interacting with receptors^58^, being required to conform its structure for an effective binding.

Moreover, in this study we detected a discrepancy between apoferritin and insulin in the effect of hydration water onto the low temperature mobility of the protein. The absence of a dry/hydrated dynamical inversion in insulin, as found for other proteins, will be a subject of further investigation with both experiments and simulations.

Looking forward, interesting *addendums* to this work include (i) exploring the behaviour of proteins in water-depleted environment at denaturing temperature conditions^59,60^ and (ii) the prospect to investigate reversibility of structural changes induced by lyophilisation; both apoferritin and insulin being typically stored in lyophilised form and reconstituted in solution for laboratory uses, for example, in the case of apoferritin, in protein chromatography.

For the former (i), although water plays a pivotal role for the structural unicity of the native state, the thermal denaturation temperature was found to increase in environments with a low water content, which is apparently a contradiction and needs clarification. Regarding the latter (ii), the reproduction of structural reversibility by simulations is hard, since the kinetics of secondary structure recovery is much slower than that of secondary structure degradation. In addition, being that the secondary structure needs to recover following solubilisation^61^, in case of apoferritin the simulation model would need to contain a huge number of atoms, including water molecules that fill the cavity, making the computing cost extremely high.

Nonetheless, the possibility and potential of using simulation trajectories to effectively reproduce experimental results makes ‘virtual’ neutron scattering experiments in yet unexplored conditions an exciting reality.

## Methods

### Molecular dynamics simulation

Atomistic molecular dynamics simulations of lyophilised and weakly hydrated horse spleen apoferritin and pancreatic bovine insulin were performed. Detailed descriptions of model systems construction, simulation conditions and procedures for the trajectory extraction and analysis are reported in the Supplementary Information (see Supplementary Methods 1 and Supplementary Methods 2).

**Fig. 10:**
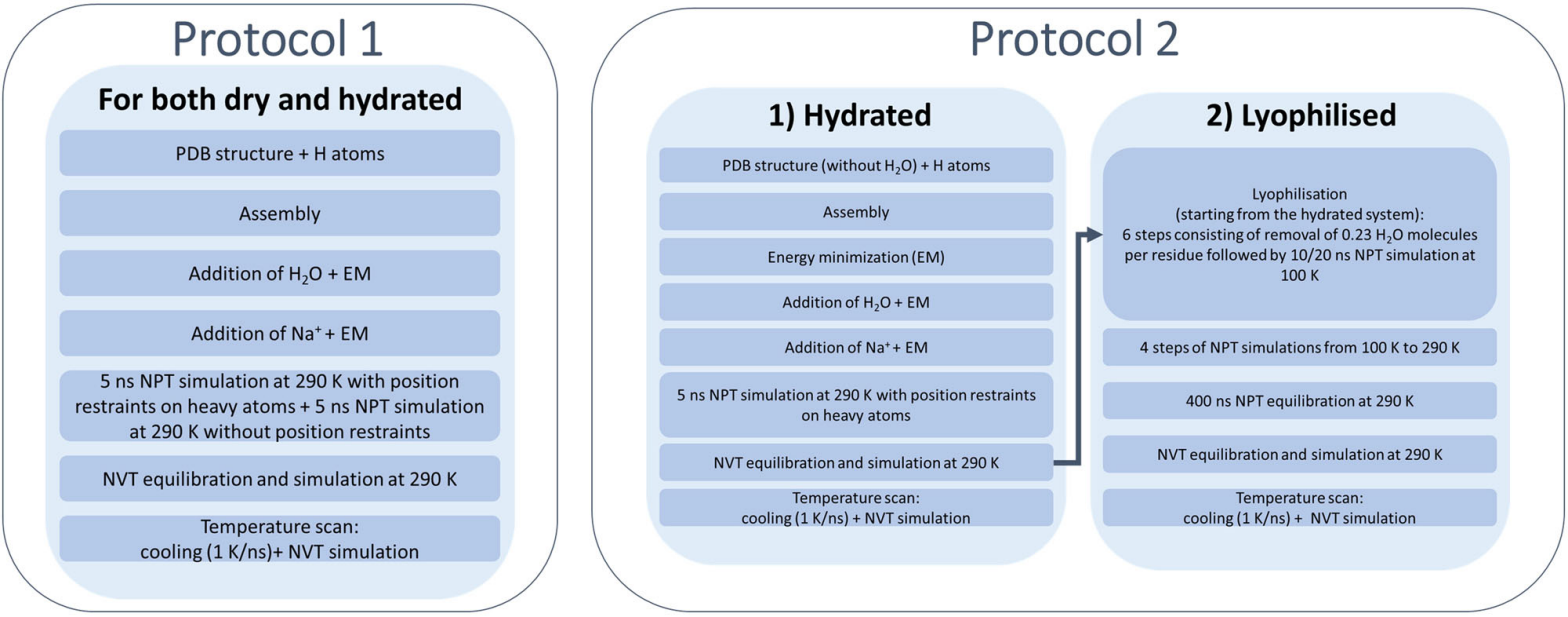
Schematic description of the two simulation protocol computing steps. EM energy minimisation.

Two simulation protocols were used to build lyophilised and weakly hydrated apoferritin assemblies with the aim of developing an in-silico lyophilisation method more representative of that used for in vitro neutron sample preparation. The two protocols (Protocol 1 and Protocol 2) are described in the schematic (Fig. 10). The first follows the more routinely adopted procedure^6,12–16^ for generating dehydrated or weakly hydrated models. The second (Protocol 2), considers a refined approach in which the molecular packing of the hydrated system is obtained by a milder equilibration procedure and an actual sample preparation lyophilisation process is mimicked. As shown, dynamical analysis of those simulations created using Protocol 2 aligns best with neutron experiments.

In terms of hydration level, we assumed a water content for the dry models of *h* = 0.05 (*h* = gram of D_2_O per gram of protein), according to the level of hydration expected for lyophilised materials after primary drying^62^. The hydrated apoferritin models were prepared with *h* = 0.31, according to the hydration level of sample used for the neutron experiment. Highlighting the *h* value, the models are termed apo_h005 and apo_h031 for the dry and hydrated protein, respectively. Consistently to the experiments, the weakly hydrated insulin model was prepared with *h* = 0.25. Simulated models for insulin are termed ins_h005 and ins_h025, referring to the lyophilised and hydrated states, respectively. The detailed composition of the systems is reported in Table 1.

**Table 1:** Composition and density of the simulated systems

	apo		ins	
	h005	h031	h005	h025
Subunits	24	24	2	2
Residues per subunit	170	170	51	51
Biological assembly	24-mer	24-mer	2-mer	2-mer
Water molecules	1257	6844	29	145
NA ions	168	168	4	4
Total number of atoms	69051	85812	1641	1989
Density (g dm^−3^)	722.6	532.5	960.2	803.2

Considering Protocol 2 first, hydrated models were built with a softer equilibration process, missing the NPT step without position restraints of Protocol 1, and lyophilised models were generated with a specific route, going from the hydrated system to the dry one, as illustrated in Fig. 10. These procedures, where a more gradual rearrangement of the molecular packing in the hydrated system is allowed and the primary drying step of an in vitro freeze-drying process is mimicked, lead to a more realistic description of systems.

The biological assembly of apoferritin (24-mer) was obtained from the Protein Data Bank crystal structure 2W0O^63^. It should be noted that, because of its large native size, the system was modelled as a single biological assembly. The adoption of an isotropic model including multiple apoferritin biological assemblies would increase the number of atoms of about one order of magnitude, with a considerable increase of computing effort. As will be shown, the efficacy of this seemingly minimalistic choice was demonstrated a posteriori by the favourable experiment-simulation agreement. This also indirectly suggests that the success of such a simulation approach does not need extraordinary computing facilities.

Insulin was simulated in its dimeric form (MW = 11555.07 Da), using as a reference the biological assembly 2 of the Zn-free insulin crystal structure 1APH^64^.

For the weakly hydrated protein models, after density equilibration at 290 K in an NPT environment by constraining the position of non-hydrogen protein atoms, we moved to NVT conditions where structure equilibration was achieved. Trajectories were acquired at 16 distinct temperatures. The simulation temperature scan was guided by the experimental data temperature points and performed on cooling from room temperature to 10 K at 1 K ns^−1^. Isothermal runs of about 180 ns were collected, with a sampling frequency of 1 frame every 5 ps. At 290 K, the NVT run of both the hydrated and dry model of apoferritin was prolonged to 550 ns. This was to evaluate any possible influence of simulation length on simulated apoferritin properties. Analysis of the secondary structure (see Supplementary Methods 2.1 and Supplementary Note 3), and calculation of msd, both by the software package, MDANSE^65^, as explained later, and directly from the trajectory files, was performed over two different NVT run time regimes using trajectories with length of about 200 ns and 550 ns; the last two contiguous 10 ns trajectory segments being considered for each time regime. Supplementary Table 3 reports the results, expressed as an average value and standard deviation of parameters extracted from the last two 10 ns segments. The discrepancy between the values obtained in the two different temporal regimes is within 11%, and for the majority of the observables is lower than 4%. This check demonstrates no untoward effect of simulation length on detected parameters and gives us confidence in the analysis of trajectories up to 200 ns for system characterisation. Moreover, the reproducibility of the results has been verified in an independent simulation replica, as shown in Supplementary Table 3.

For insulin models, we performed 12 independent simulations for each temperature and the reported results and errors are the average and standard deviation from all the replicas, when not differently specified.

The equilibration condition for the molecular structure in each trajectory was verified by monitoring the long time/plateau behaviour of the Root Mean Squared Deviation of the protein’s atoms. The last two 10 ns intervals were typically considered for analyses. It should be noted that no Centre of Mass correction was applied to the trajectory files used for dynamical analysis for the reasons given in the SI (see Supplementary Methods 1.3).

As stated, Protocol 2 was developed to obtain a realistic representation of the molecular packing of the weakly hydrated and lyophilised systems. At this aim, we adopted a mild equilibration procedure for the hydrated system and an in-silico dehydration of the hydrated system which mimics the lyophilisation process. However, to date, most routinely adopted procedures^6,12–16^ for generating dehydrated or weakly hydrated models follow Protocol 1; the starting configuration of the NVT simulation at 290 K being built independently and with a common equilibration procedure for the hydrated and dry models by adding different amounts of water to the same initial structure. Using the approach of Protocol 1, corresponding series of apoferritin simulations were therefore generated for comparison with those generated using Protocol 2.

MD simulations were performed with the GROMACS software package^66^ (version 2020.6), using the OPLS-AA force field^36^ and the TIP3P water model^35^. A number of TIP3P water molecules, equal to the number of D_2_O molecules present in the hydration levels of interest, were added to the simulated systems. All bonds involving hydrogen atoms were constrained using the LINCS procedure^67^ allowing a simulation time step of 2 fs with the leapfrog integration algorithm^68^.

### The apoferritin and insulin molecules

MD simulations of the iron storage protein apoferritin and the globular protein insulin in both the lyophilised and weakly hydrated state were performed as a function of temperature. The resulting simulated model assemblies were validated (dynamically) by comparing the temperature dependence of the mean squared displacement parameters extracted from simulated (MSD(T)) and experimental, <u^2^(T)>, neutron scattering data.

Iron is an element of primary importance for the metabolism and growth of virtually all living organisms. However, any un-complexed iron reacting with superoxide generates hydroxyl radicals which can cause extensive oxidative damage to proteins, lipids and nucleic acids. Sequestration of iron in a soluble, bio-available and non-toxic form is therefore essential and is accomplished by encapsulating the metal ions, (FeOOH)_8_(FeO:OPO_3_H_2_), in a specialised, spherical protein whose holo- form is named ferritin^69^. The iron-depleted form of ferritin, apoferritin, is stable in vitro and consists of a hollow pseudo-spherical non-covalent assembly, permeable to both water and small molecules/ions^70^. To date, the best-characterised mammalian iron storage protein is horse spleen ferritin^71^. Horse spleen apoferritin (Sigma-Aldrich, product no. A3641) was used for the neutron scattering experiments referred to here and to which the MD results are likened. For this work, the PDB structure 2W0O^63^ of the apoferritin shell associated with horse spleen ferritin (Fig. 1a) was used.

Horse spleen apoferritin has a molecular weight (MW) of about 444,000 Da and is an oligomer composed of 24 identical polypeptide chains, each having a MW of 18,500 Da^71^. At concentrations as low as 0.01 µM there is no evidence of subunit dissociation^18^. The subunits are arranged in a 432-point symmetry to form a nearly spherical hollow shell with outside and inside diameters ~130 Å and 75 Å, respectively^69^. Apoferritin catalyses the oxidation of the Fe(II) retained inside the cavity, and is large enough to store up to 4500 Fe atoms. The α-helix conformation is preponderant and inter-subunit channels provide an access route for the Fe atoms^69,70^. Apoferritin subunit has a total charge of −7 at pH = 7^23^. The method of preparing horse spleen apoferritin for the neutron scattering experiments is outlined in detail in the Supplementary Information (Supplementary Methods 4).

In contrast, insulin is a low molecular weight (5800 Da^20^) globular protein and representative of a family of proteins that are evolutionarily related and have in common the structural feature of disulfide bonds^72^. It consists of two polypeptide chains, A- and B- chains, of 21 and 30 residues, respectively, linked by two interchain disulfide bonds, with an intra-chain disulfide bond in the A-chain^73^. Insulin regulates the uptake and storage of glucose in the liver and muscles as well as the biosynthesis of triglycerides by fat cells and the human protein differs from bovine insulin, here considered, at three amino acid positions^73^ .

### Quasi-elastic neutron scattering and the elastic fixed window scan method

QENS is a valuable tool for the study of atomic motions in bio- materials^74,31^. Since quasi-elastic neutron scattering, and the associated elastic fixed window scan analysis method, are well-established experimental techniques, and described in detail elsewhere^19^ only a summary pertinent to this work is given here.

To summarise, QENS allows low-energy (typically ± 2000 micro electron-volts (μeV)) collective or self- motions of molecules and/or atom types to be investigated; dynamic phenomena which may be thought of as diffusive in nature. When we think of diffusive here we might focus on processes that are driven by atoms or molecules moving freely through a medium or alternatively, as in the case of a protein, along a more constrained path such that they can move only within a specific volume. Depending on the neutron instrumental setup, the length scale that can be probed using QENS ranges from ~1 Å to ~500 Å, thus covering both inter and intra molecular distances, while the timescale of the motions that can be studied spans ~1 ps to ~1000 ns.

The QENS spectrometers used to collect the experimental data presented in this work operate in time-of-flight mode. Here changes in neutron energy incurred during a neutron-nucleus collision are determined by recording the time needed for an incident neutron to travel from source to detector. A plot of detected neutron counts vs. time in any detector will resemble a peak of intensity I, of width (full width at half maximum, f.w.h.m) Γ, centred about the energy transferred, Δ*ħω*, = 0, or elastic scattering, position. It is by modelling the width and height of this peak as a function of scattering angle that information about the time scale and geometry of molecular motion(s) in the sample can be ascertained. For any sample, the variation of I and Γ as a function of scattering angle is unique and embodied in a construct known as the Scattering Function, *S*(*Q*,*ω*).

Experimentally, elastic fixed window scan (EFWS) measurements provide information on the geometry and frequency of localised modes as well as a measure of the mean square displacement, <u^2^(T)>, of specific atom types or groups. Information about the elastic scattering process alone can be ascertained by counting only those neutrons scattered within a narrow energy interval about the elastic line, Δ*ħω* = 0.

Due to the large incoherent neutron scattering cross-section of the ^1^H nucleus, the EFWS analysis method, when used to study hydrogen rich materials, is predominantly associated with the incoherent scattering function, S_i_(*Q*,*ω*), with the recorded intensity at the elastic line intensity being denoted S_i_(*Q*, *ω* ∼ 0,Τ) = I^el^_i_(*Q*,*T*); where *Q* and *ω* are the momentum and energy transfer respectively. It should be noted that since D_2_O was used to hydrate the neutron sample, the contribution to the scattering function from the D_2_O molecule could be neglected during analysis for the reasons given in the Supplementary Information (see Supplementary Note 7).

Since hydrogen atoms are abundant, and widely distributed, in proteins, this neutron method provides a global view of internal protein dynamics. As a result, incoherent neutron scattering is able to experimentally corroborate atomistic and molecular dynamics simulations; the quantity measured during an incoherent scattering experiment, S_i_(*Q*,*ω*), contains the Fourier transform of the self-correlation function computed in simulations.

Structural integrity can be gauged by extracting the mean squared displacement parameter from the EFWS data; a parameter which corresponds to the average harmonic displacement amplitude of all atomic motions in the sample. If the elastic scattering intensity, at any given temperature, is monitored as a function of *Q* then an effective (all atom) <u^2^(T)> parameter can be obtained by fitting detected neutron intensity at Δ*ħω* ~ 0 to,

2 $${I}_{i}^{{el}}\left(Q,T\right)={I}_{o}^{{el}}\left({{{{{\rm{Q}}}}}},{{{{{{\rm{T}}}}}}}_{{base}}\right)\cdot \exp \left(-\frac{{Q}^{2} < {u}^{2}(T) > }{3}\right)$$

One should recognise Eq. (2) as the Debye-Waller (DW) form. Plotting <u^2^(T)>, as a function of temperature, therefore, allows structural integrity to be appraised via deviations from this purely Debye-like harmonic response at low temperatures. Such an analysis approach is now used routinely in many areas of science, in particular its leading contribution to the understanding of the interplay of hydration and internal bio-material dynamics^31,75^.

The method of extracting <u^2^(T)> itself exhibits potential limitations. First, by rearranging Eq. (2), we see that,

3 $${{{{\mathrm{ln}}}}}({I}_{i}^{{el}}\left(Q,T\right)/{I}_{o}^{{el}}({{{{{\rm{Q}}}}}},{{{{{{\rm{T}}}}}}}_{{base}}))=\left(-\frac{{Q}^{2} < {u}^{2}(T) > }{3}\right)$$

Assuming purely harmonic motion, experimental data plotted in this fashion should exhibit a linear *Q*^2^ dependence whose gradient is proportional to <u^2^(T)>. One can also appreciate that the y-axis intercept, I^el^_i_(*Q* = 0,T_base_), of the fitted data should be temperature independent. In practice, however, this may not be the case. Poor statistics, limited momentum transfer information at low *Q*, coarse detector/*Q* coverage, possible coherent scattering contamination and multiple scattering effects (especially at small *Q* values) can alter detected intensity and skew the gradient. Second, the efficacy of Eq. (3) to fully describe the *Q* dependence of the elastic intensity is limited to situations where *Q*^*2*^ < *u*^*2*^*(T)>* « 1; the so-called Gaussian Approximation (GA) introduced by ref. ^76^. Should an-harmonic processes, such as heterogeneity of motion due to additional degrees of freedom, become evident, then Eq. (3) no longer provides a faithful description of the data at all *Q* values and modifications are needed^41,77^.

Spectra were collected using the backscattering spectrometers OSIRIS^24^ (apoferritin) and IRIS^25^ (insulin) at the ISIS Facility, Rutherford Appleton Laboratory, U.K. The spectrometers were configured to energy analyse the scattered neutron beam using the 002 pyrolytic graphite analyser reflection (PG002) which affords a f.w.h.m energy resolution of Δ*E* = 24.5 μeV (OSIRIS) and 17.5 μeV (IRIS). Such energy resolutions equate to an upper temporal observation limit of ~150 ps. This instruments also allows access to a common momentum range 0.4–1.8 Å^−1^ which in real space equates to an effective spatial range of ~3.5–15.2 Å. As mentioned, during an EFWS measurement only those neutrons scattered by the sample with zero change of energy, or Δ*E* = 0, are considered for analysis. The energy of each elastically scattered, and thus detected, neutron is governed by the analysing crystal used for energy analysis; namely 1.845 milli-electronvolts (meV) for PG(002).

The samples were cooled using a standard orange cryostat. Data was collected upon warming from 5 to 290 K in small temperature increments (typically Δ*T* = 5 K). A spectrum from a vanadium standard at room temperature was also collected for detector calibration/efficiency purposes. The temperature dependence of the measured elastic neutron scattering intensity, i.e. I^el^_i_(Q,T,ω ~ 0) was normalised to the intensity recorded at base temperature, i.e. I^el^_i_(*Q* = 0,T = 10 K,ω ~0). The data was analysed using programmes provided by the neutron facility; namely Mantid (ISIS)^78^.

The experimental mean squared displacement parameters, <u^2^(T)>, presented were calculated from the QENS data by modelling the variation of the elastic neutron scattering intensity, as a function of momentum transfer, *Q* (in this work from 0.4 to 1.8 Å^−1^) and *T* (from 10 to 290 K) according to Eq. (3). For this analysis, *I*_o_ was allowed to float, given limited *Q* information below *Q* = 0.4 Å^−1^ on the two instruments and for the more expansive reasons given in ref. ^74^. In addition, to improve statistics, the experimental elastic scattering intensity was determined by integrating neutron counts at each temperature between Δ*ħω*_min_ = −*Γ*_res_/2 and Δ*ħω*_max_ = +*Γ*_res_/2, rather than just logging peak height at Δ*ħω* = 0. For OSIRIS, this integration range was set to ±12.5 μeV; a range less than the f.w.h.m of the instrument resolution function to ensure only elastically scattered neutrons were counted. For IRIS, this range was ±8.5 μeV.

### MDANSE^65^: MD trajectory elaboration to replicate neutron scattering data

By using the MD simulation trajectory analysis package, MDANSE^65^, and by converting the MD simulation trajectory files to Dynamic Incoherent Structure Factor (DISF), S_i_(*Q*,*ω*), data, the exact same msd(T) analysis described above could be performed on the simulated data and compared directly to the experimental results. Information about MDANSE analysis and associated Python scripting files used to generate the DISF data are given in the Supplementary Information (see Supplementary Methods 3).

A couple of comments about the trajectory conversion and analysis. First, DISF generation needs the neutron instrument’s resolution function to be defined. MDANSE offers generic resolution forms (Gaussian, Lorentzian, Pseudo-Voight (PV) etc.). However, these do not accurately describe the more complex resolution function of the OSIRIS and IRIS instruments; arising from the ISIS pulse structure, neutron moderator characteristics and thermal diffuse scattering from the pyrolytic graphite energy analyser crystals. However, a reliable approximation could be generated by accurately modelling experimental resolution function data above the f.w.h.m. This was most accurately achieved using the PV form. The PV parameters used to subsequently represent the instrument resolutions during conversion are listed in the python scripting files shown in the Supplementary Information (Supplementary Methods 3). Second, due to the error-free nature of the DISF information generated by MDANSE from MD trajectory data, simulated msd(T) values were calculated by modelling I^el^_i_(*Q* = 0, *T,ω* = 0) i.e. the intensity logged at Δ*ħω* = 0 rather than collating data between Δ*ħω*_min_ = –*Γ*_res_/2 and Δ*ħω*_max_ = +*Γ*_res_/2. As such, resulting msd values were also error free. Variance was gauged by analysing two comparable trajectory files (i.e., the last 10 ns and the second-to-last 10 ns of each NVT trajectory) with blocking analysis^79^ being used to generate representative errors.

### Supplementary information

Supplementary Information

Description of Additional Supplementary Files

Supplementary Data 1

Supplementary Code 1

## Data Availability

All data supporting the findings and conclusions of this study are present in the paper and/or the Supplementary Information. Numerical source data in the main manuscript is available in the file Supplementary Data 1.

## Code availability

The script reported, and described in full, in Supplementary Methods 3: MDANSE (Molecular Dynamics Analysis of Neutron Scattering Experiments) is uploaded as a Supplementary code 1.
