# Supplementary material for: An experimentally representative in-silico protocol for dynamical studies of lyophilised and weakly hydrated amorphous proteins
Source: Commun Chem. 2024 Apr 12;7:83. doi: 10.1038/s42004-024-01167-6 (PMC11014950; doi:10.1038/s42004-024-01167-6)
Supplement: Supplementary file 1 — Supplementary Information [file 42004_2024_1167_MOESM1_ESM.pdf]

Supplementary Information (SI)

An experimentally representative in silico protocol for dynamical studies of lyophilized and weakly hydrated amorphous proteins

Elisa Bassotti, Sara Gabrielli, Gaio Paradossi, Ester Chiessi* and Mark Telling*

* Corresponding authors. Email: mark.telling@stfc.ac.uk, ester.chiessi@uniroma.it

Sections

Supplementary Methods

Supplementary Methods 1: Simulation Procedures

- Supplementary Methods 1.1: Building Model Systems and Temperature Scans
  - Supplementary Methods 1.1.1: Protocol 2: Weakly Hydrated Apoferritin, apo_h031
  - Supplementary Methods 1.1.2: Protocol 2: Lyophilised Apoferritin, apo_h005
  - Supplementary Methods 1.1.3: Protocol 1: Lyophilised (apo_h005) and Hydrated (apo_h031) Apoferritin
  - Supplementary Methods 1.1.4: Protocol 2: Lyophilised (ins_h005) and Hydrated (ins_h025) Insulin
- Supplementary Methods 1.2: Computing Details, Trajectory Acquisition and Processing
- Supplementary Methods 1.3: Centre of Mass Motion (COM) of the biological assembly

Supplementary Methods 2: Trajectory Analyses

- Supplementary Methods 2.1: Secondary Structure Analysis
- Supplementary Methods 2.2: Radial Distribution Function (RDF) Analysis
- Supplementary Methods 2.3: Mean Squared Displacement analysis in the direct space, MSD(t)
- Supplementary Methods 2.4: Computational Observables of Hydration Water Mobility
- Supplementary Methods 2.5: Influence of Simulation Length on Apoferritin Properties and Reproducibility

Supplementary Methods 3: MDANSE (Molecular Dynamics Analysis of Neutron Scattering Experiments)

Supplementary Methods 4: The Apoferritin and Insulin Molecules: Neutron Experiment Sample Preparation

Supplementary Notes

Supplementary Note 1: Comparison between Protocol 1 and Protocol 2 Results for Apoferritin

Supplementary Note 2: Comparison between OPLS-AA and CHARMMv27 force fields

Supplementary Note 3: Secondary Structure of Apoferritin

Supplementary Note 4: Hydration of methyl-containing and non-methyl-containing residues in apo_h031 at 150 K

Supplementary Note 5: Hydration of residues in ins_h025 at 290 K

Supplementary Note 6: Non-rotational Mean Squared Displacement of methyl hydrogen atoms in apo_h031 and ins_h025

Supplementary Note 7: The Scattering Cross section and the importance of the hydrogen atom

Supplementary Note 8: Simulated Effect of H/D Exchange in Weakly Hydrated Apoferritin

Supplementary Methods 1: Simulation Procedures

Supplementary Methods 1.1: Building Model Systems and Temperature Scans

To date, most simulative methodologies for generating model lyophilised or weakly hydrated (sub-monolayer coverage) systems have focused on the addition of water to the same initial starting structure and a subsequent common equilibration procedure [1-5] [6] ; herein known as Protocol 1. In this paper, however, we propose an alternative, more representative *in-silico* protocol for generating weakly hydrated and corresponding lyophilised protein models. The peculiarity of our approach consists in allowing for a more gradual structural equilibration of the molecular packing of the hydrated system and in building the lyophilised model directly from the hydrated model assembly; herein referred to as Protocol 2. The two protocols (Protocol 1 and Protocol 2) are described schematically in Figure 10 of the main text and discussed in detail in the following sections.

Supplementary Methods 1.1.1:

Protocol 2: Weakly Hydrated Apoferritin, apo_h031

To obtain a starting configuration for apo_h031 we chose to use a 24-chain biological assembly generated using the asymmetric unit coordinates from Protein Data Bank (PDB) apoferritin crystal structure, 2W0O [7]. It should be noted that, because of its native size, the system was modeled as a single biological assembly.

After adding hydrogen atoms and centring the orbicular assembly in a 15 x 15 x 15 nm^3 box, a layer of water was added to the protein surface such that the number of D_2O molecules supplemented corresponded to apo_h031. D_2O molecules were treated as H_2O molecules. Na^+ counter ions were added for electro-neutrality with the ionization state of the protein being that corresponding to pH 7 according to the experimental condition. Considering that the sodium ions can cross the apoferritin channels in solution [8], Na^+ was placed randomly both inside and outside the bio-molecule's cavity. Energy minimization, with a tolerance of 1000 kJ mol^{-1} nm^{-1}, was carried each time a component was added. Using the resulting assembly, we performed a 5 ns MD simulation in an NPT environment at 290 K, applying a position restraint to the non-hydrogen atoms of the protein, with a reduction of the cubic box side to 12.26792 nm. The final configuration converged upon was used as the starting structure for an NVT simulation at 290 K; performed this time without any restraints and for a total trajectory time of 150 ns. Temperature was scanned upon cooling, with the respective NVT runs adopting their starting structure from the final configuration of the previous temperature's simulation. The system was cooled at the speed of 1 K ns^{-1} and, after reaching the desired temperature, the run was continued isothermally for about 180 ns. The temperatures explored, which were chosen to match those of neutron scattering experiments, alongside the corresponding simulation times are reported in Supplementary Table 1.

Supplementary Methods 1.1.2:

Protocol 2: Lyophilised Apoferritin, apo_h005

An *in-silico* lyophilisation protocol was implemented to obtain the dry model assembly. Adopting the final configuration of the apo_h031 NVT simulation at 290 K, the system was then dehydrated in 6 distinct steps; 0.23 water molecules *per* residue being removed at each step (those furthest from the protein surface being selected for removal each time). After each water removal stage, an NPT simulation at 100 K (10 ns for the first 3 steps and 20 ns for the subsequent ones) was carried out. This approach mimicked experimental low temperature lyophilisation conditions. The number of water molecules retained in the final configuration corresponded to apo_h005.

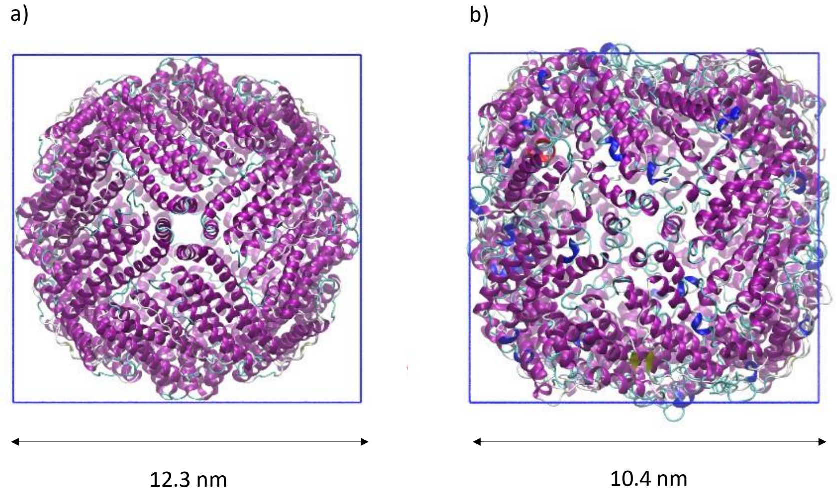

Supplementary Figure 1. a) Starting structure of apo_h031 for the NVT simulation at 290 K in the simulation box. b) Starting structure of apo_h005 for the NVT simulation at 290 K in the simulation box. α-helix, 3_{10} helix, π-helix and β-sheet regions are represented in purple, blue, red and yellow, respectively; β-turns and irregular structures are represented as cyan and white ribbons, respectively. Water molecules are omitted.

The dry apoferritin system was gradually brought from 100 to 290 K using NPT simulations, with a final equilibration in an NPT environment being carried out at 290 K for 400 ns. We subsequently moved to the NVT environment and acquired a 150 ns trajectory at 290 K. The

initial configurations of apo_h031 and apo_h005 for NVT simulation at 290 K are shown in Supplementary Figure 1a and 1b. The temperature scan was then performed with the same modality described above for the hydrated system.

	Trajectory length (ns)			
	apo		ins	
T(K)	h005	h031	h005	h025
290	150	150	200	190
280	180	180	190	190
272.5	182.5	182.5	190	190
265	182.5	182.5	190	190
257.5	182.5	182.5	190	190
250	182.5	182.5	190	190
237.5	172.13	177.5	190	190
225	187.5	187.5	190	190
200	175	175	190	190
175	175	175	190	190
150	190	190	190	190
125	180	180	190	190
100	194	180	190	190
75	175	175	190	190
50	190	190	190	190
10	200	200	190	190

Supplementary Table 1. Temperatures and simulation times (Protocol 2)

Supplementary Methods 1.1.3:

Protocol 1: Lyophilised (apo_h005) and Hydrated (apo_h031) Apoferritin

A further series of apoferritin simulations (referred to as Protocol 1 simulations) were performed. Here, the starting configuration of the NVT simulation at 290 K was built independently for the hydrated (apo_h031) and dry (apo_h005) models by adding different amounts of water to the same initial structure of the 24-mer and by performing the same subsequent equilibration procedure. Figure 10 of the main text illustrates the computing steps. The weakly hydrated model, as well as the lyophilised one, was equilibrated at 290 K by an NPT run with a position restraint of all non-hydrogen protein atoms (5 ns), and a following 5 ns NPT run without position restraints. Differently from Protocol 1, in Protocol 2 the last 5 ns NPT run is missing. The remaining conditions of the simulation are as Protocol 2, described in the preceding sections. The cubic box sides of the hydrated and dry model in the NVT runs of Protocol 1 had size of 10.50446 and 10.80655 nm, respectively.

Supplementary Methods 1.1.4:

Protocol 2: Lyophilised (ins_h005) and Hydrated (ins_h025) Insulin

By considering the aggregation propensity of bovine insulin in solution [9], we simulated insulin in its dimeric form (MW=11555.07 Da), using as a reference the biological assembly 2 of the Zn-free insulin crystal structure 1APH [10]. After centring the dimer in an orthorhombic box having size 4.4x3.4x2.4 nm^2 and adding hydrogen atoms, the protein was solvated with a number of D_2O molecules corresponding to h=0.25, to match the experimental hydration level. D_2O molecules were treated as H_2O molecules. Na^+ ions were added for electroneutrality, the total charge of insulin monomer at neutral pH being -2 [11]. Energy minimization, with a tolerance of 1000 kJ mol^{-1} nm^{-1}, was carried each time a component was added. In the crystallographic structure containing twelve dimers, the cubic box has size 7.89×7.89×7.89 nm^3, with a volume per dimer equal to 40.93 nm^3. In order to obtain a box with the same volume per dimer as in the crystallographic structure, the length of the initial orthorhombic box side was increased by a

factor of 1.04, so that the new box had size 4.576×3.536×2.496 nm^3. At the end of the 5 ns NPT simulation at 290 K with position restraints on heavy atoms the box size decreased to 4.11132×3.17693×2.24254 nm^3. The final configuration was used as the starting structure for the NVT simulation at 290 K, performed without any restraints for a total trajectory time of 190 ns.

Dehydration was carried out starting from the final configuration of the NVT trajectory at 290 K. A total of 116 water molecules were removed in 5 steps, leading to a final hydration state corresponding to h=0.05, to mimic the residual hydration after lyophilisation. Each time water molecules were removed an NPT simulation of 20 ns at 100 K was carried out. The resulting dehydrated system was then gradually heated from 100 to 290 K, with a final equilibration in an NPT environment being carried out at 290 K for 400 ns. The final configuration of the NPT equilibration at 290 K, with a box size equal to 3.66151×2.82936×1.99718 nm^3, was used as the starting structure for the 190 ns NVT simulation at 290 K. The initial configurations of ins_h025 and ins_h005 for NVT simulation at 290 K are shown in Supplementary Figure 2a and 2b. The temperature scan was then performed with the same modality described above for apoferritin. Twelve independent simulation replicas were performed for each temperature.

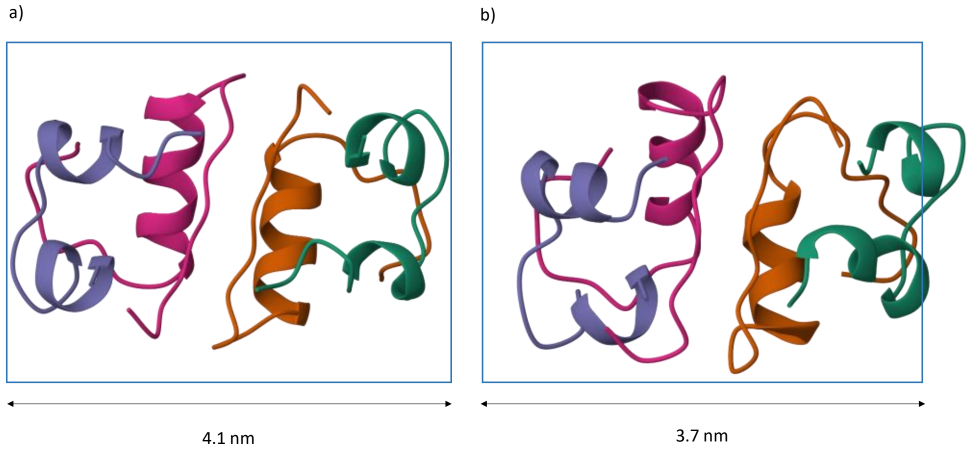

Supplementary Figure 2. a) Representation of the starting structure of ins_h025 for the NVT simulation at 290 K in the simulation box. b) Representation of the starting structure of ins_h005 for the NVT simulation at 290 K in the simulation box. Each chain is coloured differently (Chain A

is magenta in one monomer and orange in the other, Chain B is purple in one monomer and green in the other). Water molecules are omitted.

Supplementary Methods 1.2:

Computing Details, Trajectory Acquisition and Processing

MD simulations were performed using the GROMACS software package [12] (versions 2020.6), the OPLS-AA force field [13] and the TIP3P water model [14]. A number of TIP3P water molecules equal to the number of D_2O molecules present in the hydration level of interest was included into the simulated systems. All bonds involving hydrogen atoms were constrained using the LINCS procedure [15] to allow for a simulation time step of 2 fs using the leapfrog integration algorithm [16]. The Particle Mesh Ewald method[17] was used to compute long range interactions with a grid search. For short range interactions a cut-off distance of 1.0 nm was employed. Periodic boundary conditions and the minimum image convention were applied. The size of the shortest side of the simulation box for apoferritin and insulin models is equated to minimum Q-vectors of 0.06 and 0.28 Angstrom^{-1}, respectively. As such, simulations afford access to a Q-vector interval that is comparable to the experimental range used to determine the $<u^2(T)>$ values. The V-rescale thermostat[18] with a 0.1 ps time constant was used for the temperature control. The NPT simulations were run at a pressure of p=1.0 atm, using the Berendsen pressure coupling[19]. Here the time constant was set equal to 1.0 ps. The equilibration condition for the molecular structure in each trajectory was verified by monitoring the time behaviour of the Root Mean Square Deviation (RMSD) of protein heavy atoms. An NVT trajectory of about 180 ns was acquired for each system and temperature simulated, sampling 1 frame every 5 ps. The last two 10 ns intervals were typically considered for analysis.

Supplementary Methods 1.3:

Centre of Mass Motion (COM) of the biological assembly

The COM motion of the biological assembly was studied, in order to evaluate its contribution to the average mean squared displacement (MSD) value of hydrogen atoms of the protein. The X, Y, Z coordinates of the COM as a function of time were computed for the last 10 ns of the NVT trajectories at 290 K, then the time average value and standard deviation (SD) were calculated for each coordinate. The extent of the influence of COM motion on MSD was then estimated by considering the sum of SD^2, with respect to the average mobility of protein atoms, as in Supplementary Equation 1:

$$influence\ of\ COM\ motion\ on\ MSD\ (\%) = \frac{SD_x^2+SD_y^2+SD_z^2}{MSD} \cdot 100 \qquad \textit{(Supplementary Equation 1)}$$

where MSD is the average mean squared displacement value of protein hydrogen atoms at 290 K, computed as described in Section Supplementary Methods 2.3. The results are shown in Supplementary Table 2. Being the influence of COM motion on MSD ≤ 2 %, the correction for COM motion was considered not to be necessary.

	apoferritin		insulin	
	h031	h005	h025	h005
$SD_x^2+SD_y^2+SD_z^2$ (nm^2)	0.0003	0.00004	0.0007	0.0002
MSD (nm^2)	0.0262	0.01820	0.0294	0.0149
Influence (%)	1.2	0.2	2.4	1.4

Supplementary Table 2: Results of the COM motion analysis.

Supplementary Methods 2: Trajectory Analyses

The trajectory analyses were performed with GROMACS software tools and in-house packages. Graphic visualization was obtained using the molecular viewer software package VMD [20] and the web-based open-source molecular viewer Mol* Viewer [21]. Details on the analysis of specific properties are reported below.

Supplementary Methods 2.1: Secondary Structure Analysis

The time evolution of the protein's secondary structure was computed using the *DSSP* (*Define Secondary Structure of Proteins*) program [22]. In the proteins of interest β-sheet, α-helix, and 3_{10}-helix are the most populated conformational states. The percentage composition of each type was determined for apoferritin using the last 10 ns of the NVT trajectories at 50, 150 and 290 K, both in the hydrated and in the dehydrated state. The percentage composition of conformational states at 290 K was determined also for the last 10 ns of the trajectory prolonged up to 550 ns, and for a corresponding simulation replica, validating the results obtained after a shorter simulation time (Supplementary Table 3).

Supplementary Methods 2.2: Radial Distribution Function (RDF) Analysis

The radial distribution of a specie, or species, around a reference position is determined using the Radial Distribution Function (RDF):

$$RDF(r) = \frac{\langle \rho(r) \rangle}{\langle \rho_{local} \rangle} \qquad \textit{(Supplementary Equation 2)}$$

Here $\langle \rho(r) \rangle$ is the density of the specie(s) at a spherical distance *r* from a point of reference and $\langle \rho_{local} \rangle$ is the density of said specie(s) in a sphere centered at the reference position itself (radius r_{max} corresponding to half the box length). The reference position can either be that of a class of atoms or the center of mass (COM) of a group of atoms. Hydrogen atoms are neglected when

calculating some kinds of RDFs because the position of hydrogen atoms is actually defined by bond and angle geometry requirements and is therefore determined by the position of non-hydrogen (heavy) atoms. For the work presented here, RDF analysis was used to identify spatial distribution of structural components in apoferritin; the system being characterized in terms of the RDFs for non-hydrogen protein atoms, water molecules, and sodium ions around the protein center of mass. RDFs for heavy atoms of apo_h031, apo_h005 (calculated using the last 10 ns of the 290 K NVT trajectory) and of the crystallographic structure were compared. In addition, RDFs with respect to the center of mass of the protein were computed separately for methyl-containing and non-methyl-containing residue groups, in order to achieve better understanding on how the locality of these specific groups aligned with water molecules. These latter RDFs were calculated using the last 10 ns of the apo_h031 NVT trajectories at 290 K and 150 K and including all atoms of the residue. Finally, RDFs with respect to the center of mass of apoferritin were computed for heavy atoms of selected groups of residues of apo_h031 at 290 K based on the influence that hydration exerts on the residue mobility.

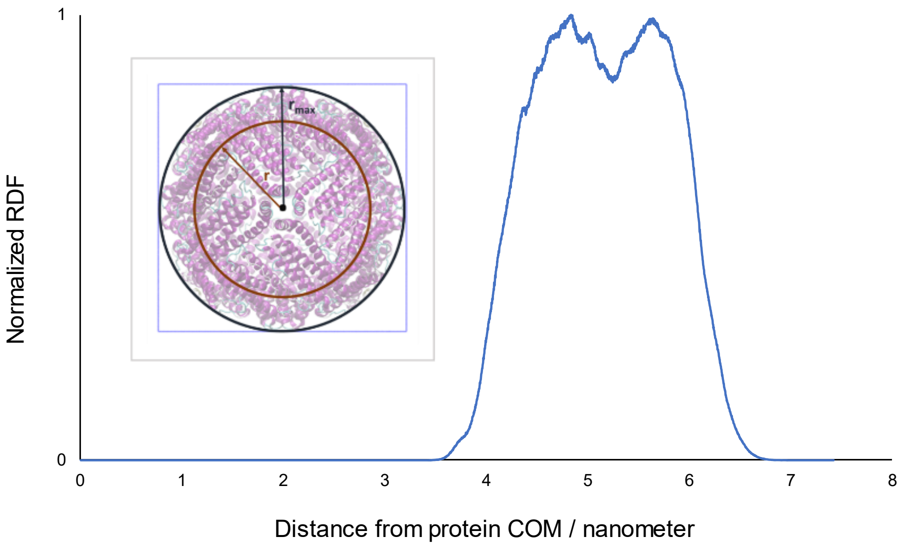

Supplementary Figure 3. Example RDF as calculated for all non-hydrogen protein atoms in apo_h031 at T = 290 K. Inset: schematic representation of how the RDF is calculated for apoferritin.

Supplementary Methods 2.3: Mean Squared Displacement analysis in the direct space, MSD(t)

The ensemble average mean square displacement (MSD) of all hydrogen atoms in the proteins of interest, and hence protein mobility, was calculated as a function of time at a given temperature using Supplementary Equation 3. Hydrogen atoms are considered alone here since scattering from this atom type dominates the signal measured experimentally.

$$MSD(t) = \langle |\boldsymbol{r(t)} - \boldsymbol{r_0}|^2 \rangle \qquad \textit{(Supplementary Equation 3)}$$

Here $\boldsymbol{r_0}$ is the reference position of the particle at time t = 0, ***r(t)*** is the position of the particle at time t and an average is performed over both time origins and hydrogen atoms. The MSD(t) calculation was carried out using both GROMACS and MDANSE [23] routines, verifying consistency. A time independent MSD value was calculated as a function of temperature using the procedure described below; MSD value deduced from direct space analysis is named $MSD_t(T)$ to distinguish it from the MSD(T) parameter obtained from the simulated neutron results. The experimental MSD parameter is named $\langle u(T)^2 \rangle$. MSD(t) values were calculated from two production run intervals, i.e., the last 10 ns and the second-to-last 10 ns of each NVT trajectory. To obtain the time independent $MSD_t(T)$ value, for each 10 ns production run the MSD(t) curves were averaged over the interval 6-8 ns (see Supplementary Figure 4(a)). In this range the MSD(t) response was seen to have broadly plateaued. The resulting mean values from the two production runs were then considered for apoferritin, using standard deviation as the error. Performing the analysis over the last 40 ns trajectory in four blocks of 10 ns does not change the final average $MSD_t(T)$ value (see Supplementary Figure 4(b)). For insulin the $MSD_t(T)$ value was obtained as the average over the twelve independent simulation replicas, using standard error as the error.

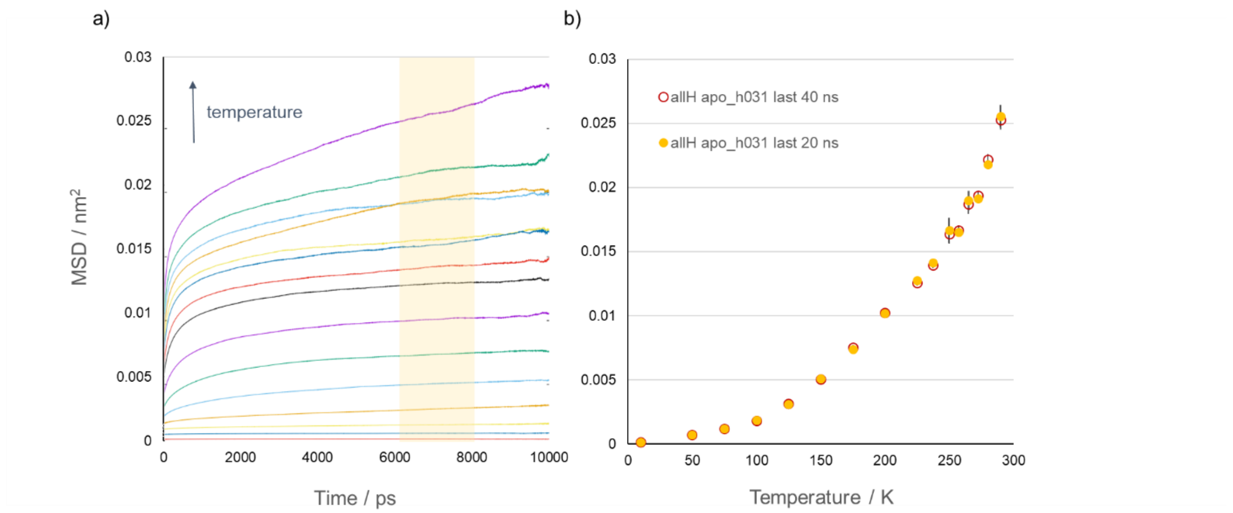

Supplementary Figure 4. a) Example of output from MSD(t) at all temperatures, calculated for all H atoms of apo_h031 in the last 10 ns of the 290 K NVT trajectory. The time range 6-8 ns, over which the MSD(t) was averaged to obtain the lone $MSD_t(T)$ value, is highlighted in yellow. b) Example of output from $MSD_t(T)$ calculated for all H atoms of apo_h031. Each lone $MSD_t(T)$ value is the average of the $MSD_t(T)$ values for i) the last two (full yellow symbols) and ii) the last four 10 ns (empty orange symbols) trajectory intervals. Errors were generated using the standard deviation.

Initially, MSD analysis was performed by considering all hydrogen atoms. This allowed the overall dynamic response of protein to be monitored. Here, labile hydrogens atoms, namely those protein hydrogen atoms that can be exchanged with water hydrogen atoms, are also included, since lyophilisation of the samples used for the neutron scattering experiments was carried out using de-ionized H_2O buffer solutions. The selection of H atoms assigned to specific groups allowed the contribution of different species to overall protein mobility to be isolated; the MSD of methyl and non-methyl hydrogen atoms, in particular, being computed using the procedure outlined above. In addition, a specific analysis of MSD for methyl hydrogen atoms has been performed, to discriminate the rotational and non-rotational contributions to the mobility of these hydrogen atoms [24]. In this case the MSD values at t=1 ns for the lyophilised and hydrated systems have been considered, for comparison to literature data [5].

The MSD of individual residues of apoferritin in the 6-8 ns time range was also determined, again including only hydrogen atoms and averaging over the 24 apoferritin subunits.

Supplementary Methods 2.4: Computational Observables of Hydration Water Mobility

The MSD vs time (Supplementary Equation 3) of water oxygen atoms (OW) for apo_h031 and ins_h025 was computed for all simulated temperatures, analysing the last 10 ns of the trajectories of the hydrated systems. For apoferritin also the second-to-last 10 ns were considered for averaging. For insulin the MSD of water oxygen atoms was computed as the average of the MSD vs time behaviour of the twelve independent replicas of the insulin dimer simulations. Supplementary Figure 5 shows the ins_h025 results at some of the simulated temperatures.

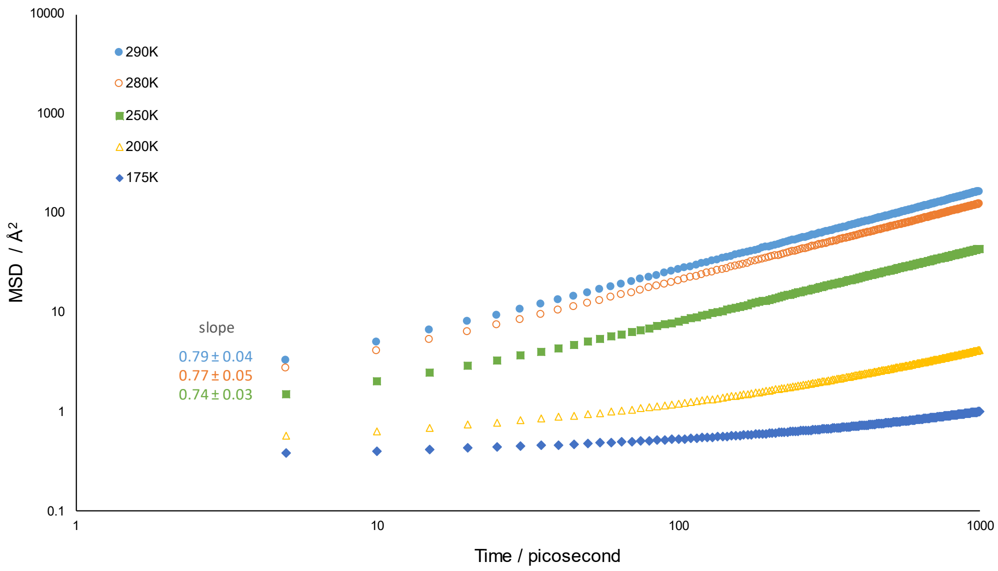

Supplementary Figure 5. MSD of water oxygen atoms in a time interval of 1 ns at 290, 280, 250, 200 and 175 K for the ins_h025 model. The exponent of the power law fit of the MSD vs time behaviour is reported for the higher temperatures.

The hydrogen bonding between water molecules of apo_h031 and ins_h025 was investigated by adopting the geometric criteria of an acceptor−donor distance lower than 0.35 nm and a hydrogen-donor−acceptor angle lower than 30°. The intermittent time autocorrelation function of water-water hydrogen bonds, $C_H(t)$, was computed in the last 20 ns trajectories and the hydrogen bond relaxation time, τ_H, was defined as the time when the corresponding autocorrelation function decays to e^{-1} (Supplementary Figure 6).

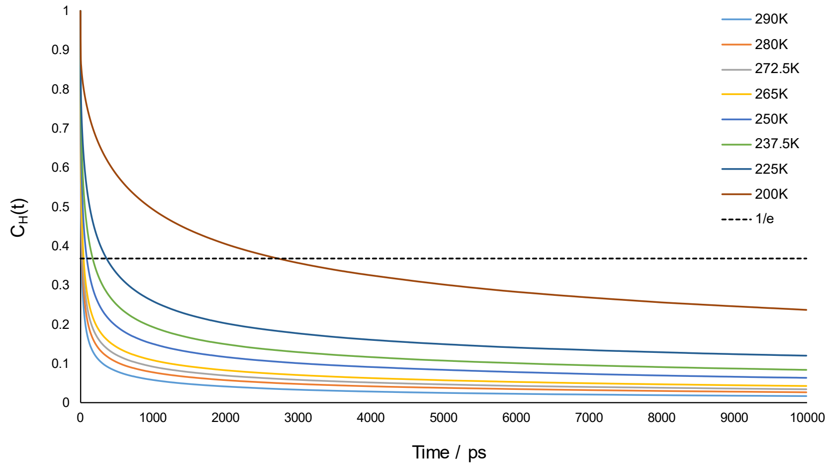

Supplementary Figure 6. Autocorrelation function, $C_H(t)$, of the water-water hydrogen bonds in apo_h031 in the temperature range 200 - 290 K. The relaxation time, τ_H, was defined as the time when the corresponding correlation function decays to e^{-1} (horizontal dashed line).

Supplementary Methods 2.5: Influence of Simulation Length on Apoferritin Properties and Reproducibility

To evaluate any possible influence of simulation length on the dynamical and structural properties being investigated, the NVT run at 290 K of both the hydrated and dry model of apoferritin was prolonged to 550 ns. Analysis of the secondary structure, and calculation of mean squared displacement, both by the software package MDANSE and directly from the trajectory files, was performed over two different NVT run time regimes using trajectories with length of

about 200 ns and 550 ns; the last two contiguous 10 ns trajectory segments being considered for each time regime. The results, in terms of average value and standard deviation of parameters extracted from the last two 10 ns segments, are reported in Supplementary Table 3. The discrepancy between the values obtained in the two different temporal regimes is within 11 %, and for the majority of the observables is lower than 4%. In addition, the reproducibility of the results has been verified in an independent simulation replica, as shown below.

	**Trajectory length (ns)**			
	**~ 200**		**550**	
	**replica 0**	**replica 1**	**replica 0**	**replica 1**
**α-helix apo_h031 (%)**	63.6 ± 0.4	62.3 ± 0.4	61.8 ± 0.4	61.7 ± 0.4
**3_{10}-helix apo_h031(%)**	1.0 ± 0.2	1.0 ± 0.2	1.1 ± 0.2	1.3 ± 0.2
**α-helix apo_h005 (%)**	49.8 ± 0.5	50.2 ± 0.5	47.6 ± 0.5	48.8± 0.5
**3_{10}-helix apo_h005(%)**	4.6 ±0.4	4.3 ±0.4	5.2 ± 0.4	4.9 ±0.4
**MSD apo_h031(Å^2)**	0.428 ± 0.001	0.421 ± 0.001	0.406 ± 0.002	0.408 ± 0.001
**MSD apo_h005(Å^2)**	0.344 ± 0.001	0.341 ± 0.001	0.33992 ± 0.00009	0.341 ± 0.001
**MSD_t apo_h031(nm^2)**	0.0256 ± 0.0009	0.0257 ± 0.0008	0.0230 ± 0.0008	0.0212 ± 0.0001
**MSD_t apo_h005(nm^2)**	0.0173 ± 0.0002	0.0172 ± 0.0001	0.01660 ± 0.00001	0.01630 ± 0.00003

Supplementary Table 3: ***Influence of simulation length on apoferritin properties and reproducibility.*** *Secondary structure compositions and MSD(T), $MSD_t(T)$ for apo_h031 and apo_h005 at T = 290 K as determined by analysing trajectories with length of about 200 ns and 550 ns. Average values and standard deviations from the last two 10 ns trajectory intervals. Results from two independent simulations (named as replica 0 and replica 1) are reported.*

Supplementary Methods 3: MDANSE (Molecular Dynamics Analysis of Neutron Scattering Experiments)

Using the MD simulation trajectory analysis package, MDANSE [23], and by converting the MD simulation trajectory files to Dynamic Incoherent Structure Factor (DISF) data, $S_{inc}(Q,\omega)$, the exact same MSD(T) analysis performed on the experimental data could be replicated. The MDANSE python scripting file used to generate the DISF data is shown below. Comments about the scripting nomenclature are included inline.

As stated in the main text, DISF generation needs the neutron instrument's resolution function to be defined. MDANSE offers generic resolution forms (Gaussian, Lorentzian, Pseudo-Voight (PV) etc.). However, these do not accurately describe the more complex resolution function of the OSIRIS instrument; arising from the ISIS pulse structure, neutron moderator characteristics and thermal diffuse scattering from the pyrolytic graphite energy analyser crystals [25]. However, a reliable approximation could be generated by accurately modelling the experimental resolution function data above the f.w.h.m using the PV form (Supplementary Figure 7). The PV parameters used to represent the instrument resolution in MDANSE are listed in the python scripting file below. A Pseudo-Voigt function is a linear combination of a Gaussian and a Lorentzian function:

$$PV(x) = (1-n)G(x) + nL(x) = A \cdot \left\{(1-n) \cdot \left[\frac{1}{\sigma_G\sqrt{2\pi}} \cdot \exp -\frac{(x-\mu_G)^2}{2\sigma_G^2}\right] + n \cdot \left[\frac{1}{\pi} \cdot \frac{\sigma_L}{(\mu_L - x)^2 + {\sigma_L}^2}\right]\right\}$$

(Supplementary Equation 4)

where A is the amplitude of the function, σ_G and μ_G are the variance and centre of the Gaussian function, σ_L is the half-width at half-maximum of the Lorentzian function and μ_L is the centre of the Lorentzian distribution. The parameter *n* (values between 0 and 1) shifts the profile of the function towards pure Gaussian or pure Lorentzian when approaching 0 or 1 respectively.

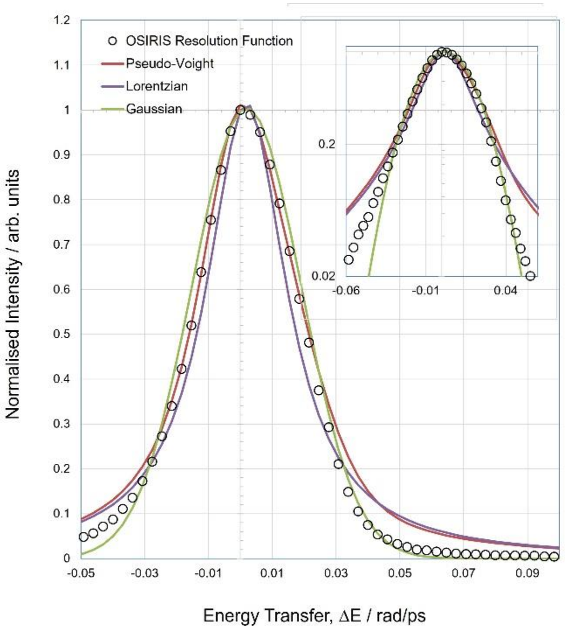

Supplementary Figure 7: Plots of the Pseudo-Voigt function (red) fitted to the experimental resolution data (hollow circles) from the OSIRIS (f.w.h.m = 24.5 μeV) instrument. The fit is compared to simple Lorentzian (purple) and Gaussian (green) function fits. The normalised detected neutron intensity is plotted against the energy transfer (ΔE) expressed in radians (rad)/pico-second (ps) (1 μeV = (6.3/4.1) x 10^{-3} rad/ps). PV fit parameters are: σ_G = 0.013947, μ_G = 0.010784, σ_L = 0.017048, μ_L = -0.001, n = 0.841868

```
############################################################
# This is an automatically generated MDANSE run script
#############################################################
from MDANSE import REGISTRY
# Your input files
input = ['2w0o_h31_NVT_10K_last10ns.nc','2w0o_h31_NVT_290K_last10ns.nc']
# Your output files
output = ['disf_2w0o_h31_NVT_10K_last10ns.nc'. 'disf_2w0o_h31_NVT_290K_last10ns.nc']
# The name of your atom selection definition
parameters = []
from shutil import copyfile
for a, b in zip(input, output):
  copyfile(a, 'Copy.nc')
  parameters = []
  parameters['atom_selection'] = [u'Protein and All H']   ; atom selection set such that only the hydrogen atoms on each of the apoferritin molecule's 24 subunits were considered
  parameters['atom_transmutation'] = None
  parameters['frames'] = (0, 2000, 1)
  parameters['grouping_level'] = u'atom' ; grouping level 'atom' chosen such that the 'mean' dynamical behaviour of all individual hydrogen atoms across all 24 subunits was output
  parameters['instrument_resolution'] =
                (u'pseudo-voigt', {'mu_lorentzian': -0.001, 'sigma_lorentzian': 0.017048, 'sigma_gaussian': 0.013947, 'eta': 0.841868, 'mu_gaussian': 0.010784}) ; parameters of OSIRIS resolution
                (u'pseudo-voigt', {'mu_lorentzian': 0.002, 'sigma_lorentzian': 0.014, 'sigma_gaussian': 0.017, 'eta': 0.88, 'mu_gaussian': -0.01}) ; parameters of IRIS resolution
  parameters['output_files'] = (b, (u'netcdf',))
  parameters['projection'] = None
  parameters['q_vectors'] = 'Q 0 to 40 step 0p5'; |Q| vectors generated from 0 - 40 nm-1, ΔQ = 0.5 nm-1. Parameters used in the MDANSE|Q| vector generation tool were ('spherical', {'width': 1.0, 'seed': 0, 'n_vectors': 1, 'shells': (0.0, 40.0, 0.5)})
  parameters['running_mode'] = ('multiprocessor', 12)
  parameters['trajectory'] = u'H:\\Copy.nc'
  parameters['weights'] = u'b_incoherent2'
  disf = REGISTRY['job']['disf']()
  disf.run(parameters,status=True)
```

Supplementary Script 1: The MDANSE python scripting file used to generate the DISF data

Supplementary Methods 4: The Apoferritin and Insulin Molecules: Neutron Experiment Sample Preparation

Two grams of equine spleen apoferritin material (0.2 µm filtered material suspended in 0.15 M sodium chloride) was purchased from Sigma-Aldrich (product no. A3641). The suspended material was extensively dialyzed against 10 mM ammonium acetate to remove non-volatile salts. The protein was then freeze-dried. The resulting tan-white powder was further dried over drying agents (first silica gel and then potassium pentoxide). A hydrated apoferritin sample with hydration level $h \sim 0.31$ g D_2O per g protein was prepared by hydrating some of the lyophilised material over D_2O (Sigma-Aldrich, purity 99.98%). D_2O was used to hydrate the neutron sample due to the fact that the contribution to the measured scattering intensity from the deuterium oxide molecule could be neglected during analysis for the reasons given in Supplementary Note 7.

Bovine pancreatic insulin powder was purchased from Sigma-Aldrich (product no. I1882). The dry and hydrated samples were prepared as described for apoferritin. The hydration level of the insulin hydrated sample was $h \sim 0.25$ g D_2O/g protein. At these hydration levels water crystallization was not deemed problematic. Hydration levels were determined from the observed mass change upon hydration. The hydrated and the lyophilised materials were sealed in separate flat-plate aluminium sample cans. Weighing the sample cans before and after the experiment showed no change in mass had occurred during the experiment. To minimize the effects of multiple scattering, the thickness of each sample was limited such that the total scattering from the sample was no greater than ~10% (beam transmission through sample ~90%).

Supplementary Note 1: Comparison between Protocol 1 and Protocol 2 Results for Apoferritin

The direct comparison of apoferritin MSD results obtained by Protocol 1 and Protocol 2 with experimental values is displayed in Supplementary Figure 8. For apo_h005 at the higher temperatures Protocol 1 overestimates the experimental values more than Protocol 2, while for

apo_h031 above 250 K Protocol 1 gives MSDs substantially lower than experimental corresponding values. Overall, Protocol 2 is more effective in reproducing the difference of the temperature behaviour between hydrated and lyophilised protein.

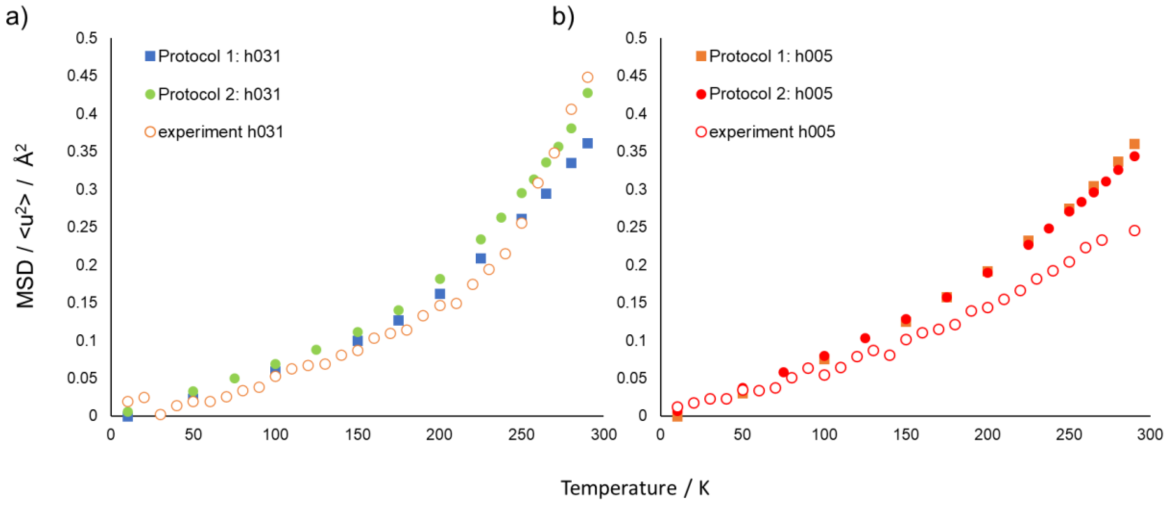

Supplementary Figure 8. Comparison of mean squared displacement parameters extracted from simulated (MSD(T)) and experimental (<u^2(T)>) neutron scattering data. a) Weakly hydrated apoferritin (Protocol 1 and Protocol 2 results are shown by full blue squares and full green circles, respectively; experimental data by empty orange circles). b) Lyophilised apoferritin (Protocol 1 and Protocol 2 results are shown by full orange squares and full red circles, respectively; experimental data by empty red circles). Where not visible, error bars are within the symbol size.

Supplementary Note 2: Comparison between OPLS-AA and CHARMMv27 force fields

At the aim to evaluate the influence of force field on the simulated neutron scattering results, we repeated the Protocol 2 simulation of apoferritin using the CHARMM v27 all-atom force field [26] and the TIP3P water model; the same force field setup as used for the simulations reported by Hong et al. [5]. Mean squared displacement (MSD) parameters extracted from simulated neutron scattering data for apo_h031 and apo_h005 using the OPLS-AA and CHARMMv27 force fields are reported in Supplementary Figure 9.

We find that the choice of force field only weakly affects apoferritin hydrogen displacement amplitudes, those obtained with CHARMMv27 being marginally lower. This small discrepancy can be ascribed to differences in the functions describing the potential energy components and in the parameters, such as partial charges, between these force fields. Nonetheless, the experiment-simulation agreement obtained with OPLS-AA and CHARMMv27 is similar.

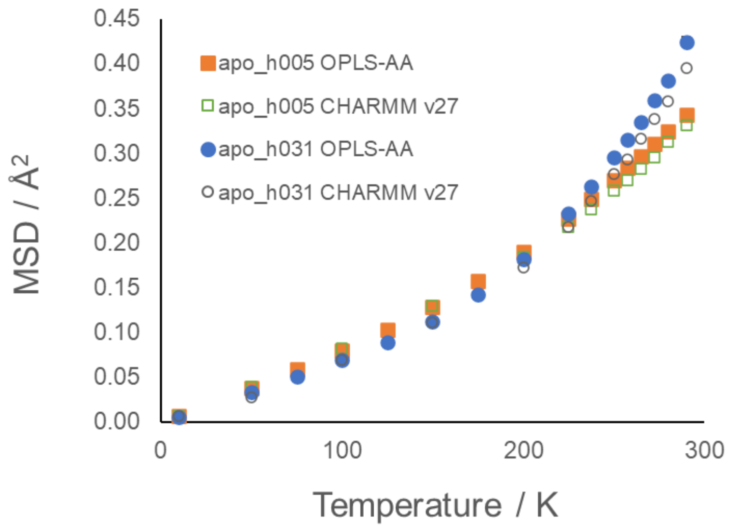

Supplementary Figure 9. Mean squared displacement parameters extracted from simulated (MSD(T)) neutron scattering data for apo_h031 (circles) and apo_h005 (squares) using Protocol 2. MSD(T) values obtained with the force field OPLS-AA or CHARMMv27 are shown in full orange or blue and empty green or black symbols, respectively. Errors of the simulated data, when not visible, are within the symbol size.

Supplementary Note 3: Secondary Structure of Apoferritin

The secondary structures of apo_h031 and apo_h005 models generated using Protocol 2 were analyzed at 50 K, 150 K and 290 K and the average percentage of each type of secondary structure component (α-helix, 3_{10}-helix and β-sheet) was calculated. This structural characterisation is suitable to demonstrate that the final simulated secondary structures, from which dynamical

information alone was obtained, remained representative of that expected crystallographically. Such acknowledgement further validates the stability of the simulated build before dynamical analysis began, especially given the complex 3D, multi component construct of the apoferritin molecule.

Little compositional variation of secondary structure was observed as a function of temperature. The results, shown in Supplementary Figure 10, were also likened to that predicted from the 2W0O crystallographic structure. Values reported in Supplementary Figure 10 indicate that hydration generally promotes the α-helix conformation. The appearance of small percentages of 3_{10}-helix, not present in the crystallographic structure, partially compensates for the loss of α-helicity in the simulated models; compensation being greatest in the lyophilised protein. The amount of β-sheet structure is negligible in both apoferritin models. These general trends agree with the experimentally observed dependence of protein conformation on water content at low hydration degrees [27]. It is worth also mentioning that, in most proteins, the structural changes caused by lyophilisation are reversible [28]. Concerning apoferritin hydration, the 2W0O crystallographic structure contains a number of crystallographic water molecules corresponding to an *h* value of 0.23 (*h* being defined as gram of D_2O per gram of protein). This crystallographic water, involved in the stabilization of the crystal packing, was removed when building the apoferritin models.

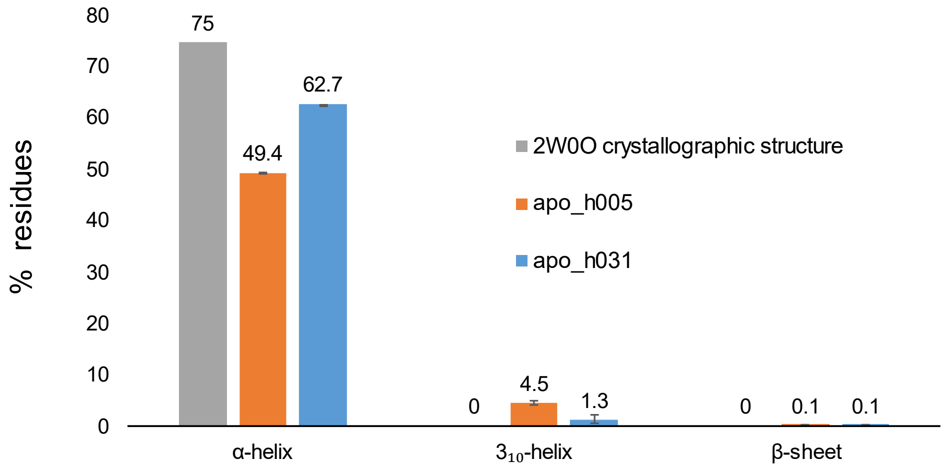

Supplementary Figure 10: Percentages of residues in α-helix, 3_{10}-helix and β-sheet conformation in i) the 2W0O crystallographic (grey) ii) apo_h005 (orange) and iii) apo_h031 (blue) structures of apoferritin. With no notable temperature dependence, these are average values and standard deviations generated by considering the percentages obtained at 50, 150 and 290 K.

Supplementary Note 4: Hydration of methyl-containing and non-methyl-containing residues in apo_h031 at 150 K

RDF analysis allowed the contribution of distinct residue sets to enhanced protein mobility to be evaluated in terms of their proximity to water. Said RDFs were computed for apo_h031 at 150 K (Supplementary Figure 11) and 290 K (Figure 7 of main text) for amino acids with, and without, methyl groups; the COM of the protein being used as the point of reference. The data was compared to the radial distribution of water molecules, as shown in Supplementary Figure 11.

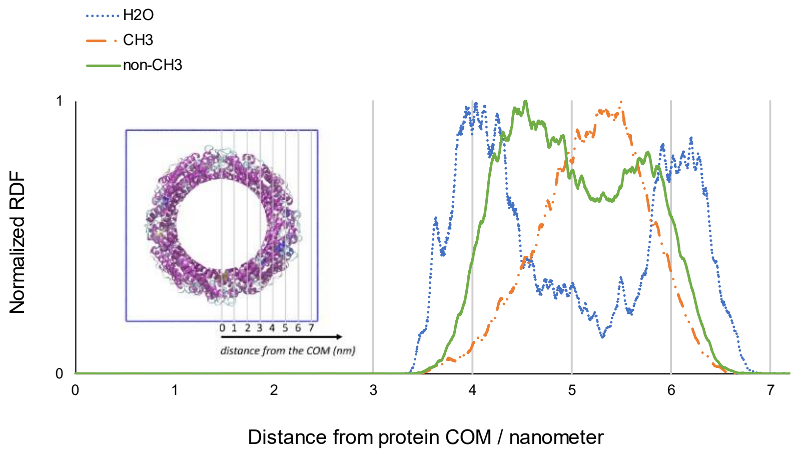

Supplementary Figure 11. RDFs of amino acids comprised of (CH_3, dash dot orange line), and not comprised of (non-CH_3, solid green line), methyl groups in apo_h031. The RDF of oxygen atoms of water molecules (h=0.31) is also shown (dotted blue line). T=150 K. The COM of the protein was chosen as the point of reference. Inset: the spatial grading, relative to the COM used in the main figure, projected onto the apo_h031 cavity.

The results at 150 K are consistent with those obtained at 290 K: amino acid species comprising of methyl groups appear to locate in protein regions less accessible to water as compared to non-methyl residues, whose distribution broadly correlates with the water molecule distribution.

Supplementary Note 5: Hydration of residues in ins_h025 at 290 K

The hydration of residues in ins_h025 at 290 K was explored by identifying the number of water molecules located within a 3.5 Å radius of each amino acid atom. In general, polar and charged amino acids (ASN, GLN, GLU, HIS, LYS) are more hydrated than average. Amino acids containing methyl groups are instead, typically, less hydrated, being the average number of hydration water molecules per residues equal to 7 (Supplementary Figure 12).

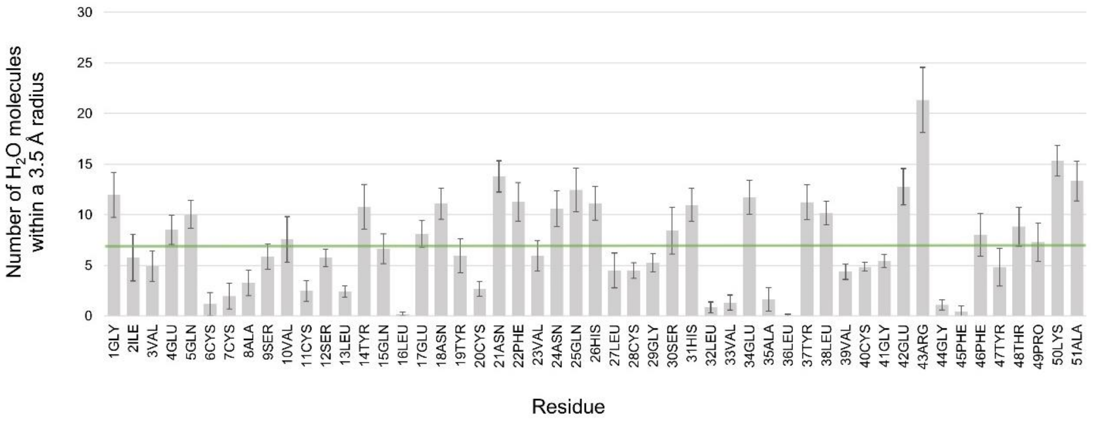

Supplementary Figure 12. Number of water molecules within a 3.5 Å radius of each amino acid atom of ins_h025 at 290 K. Error bars are standard deviations over twelve independent simulation replicas. The horizontal green line indicates the average number of water molecules per residue.

Supplementary Note 6: Non-rotational Mean Squared Displacement of methyl hydrogen atoms in apo_h031 and ins_h025

The comparison between the non-rotational component of the mean squared displacement of methyl hydrogen atoms of weakly hydrated apoferritin and insulin, apo_h031 and ins_h025, respectively, is displayed in Supplementary Figure 13. The values are obtained from direct trajectory analysis (Supplementary Equation 3) at t=1 ns. The higher mobility of insulin methyl groups at T > 100 K, as compared to apoferritin, is noteworthy.

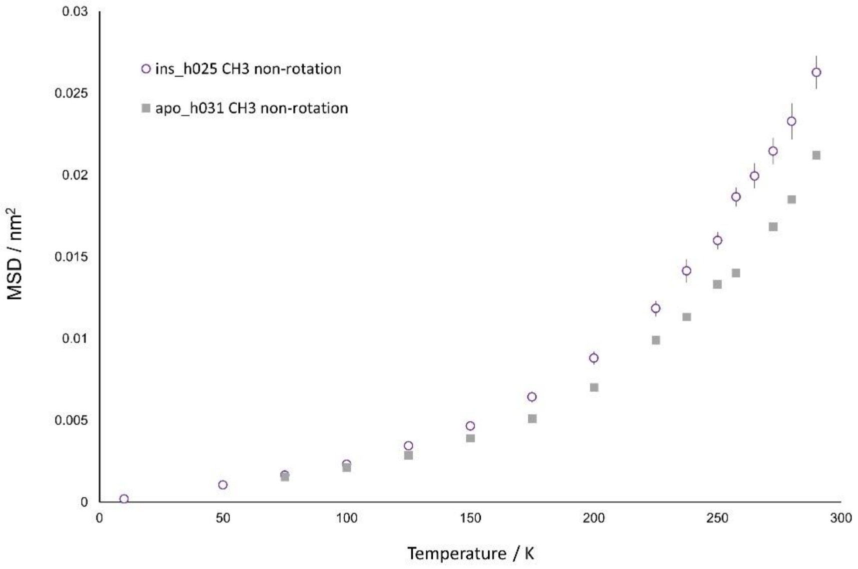

Supplementary Figure 13. Mean squared displacement at t=1 ns for non-rotational motions of methyl hydrogen atoms of apo_h031 (full grey squares) and ins_h025 (empty purple circles). Results and errors for insulin are averages and standard errors over twelve independent simulation replicas. Error bars for apoferritin are within symbol size.

Supplementary Note 7: The Scattering Cross section and the importance of the hydrogen atom

A consequence of the neutron-nucleus interaction is that a neutron can be either absorbed or scattered; the scattering process commonly considered in terms of effective areas of interaction, i.e., scattering and absorption cross sections (σ_s and σ_a). In addition, both coherent (interacting scattered neutron waves) and incoherent (non-interacting scattered neutron waves) scattering contributions are detected. However, unless a sample has been selectively labelled using deuterium, the signal measured from most hydrogen rich materials is dominated by the high incoherent scattering cross-section of the hydrogen atom ($\sigma_{H,inc} = 80.27 \times 10^{-28}\, m^2$). For comparison, the incoherent scattering cross sections of other atoms is: $\sigma_{C,inc} = 0.001 \times 10^{-28}\, m^2$, $\sigma_{N,inc} = 0.5 \times 10^{-28}\, m^2$, $\sigma_{S,inc} = 0.007 \times 10^{-28}\, m^2$, $\sigma_{O,inc} = 0.0008 \times 10^{-28}\, m^2$. As a result, any signal measured from a highly protiated material will be dominated by the

incoherent dynamic structure factor, $S_{inc}(\mathbf{Q},\omega)$, arising from scattering of ^{1}H nuclei. Since $S_{inc}(\mathbf{Q},\omega)$ describes correlations between the position of the same nucleus type at different times, the information extracted from scattering from hydrogen is dominated by the self-motion of the ^{1}H species. Because of the dominance of incoherent scattering from the H atom position in the systems studied here, the experimental signals collected were assumed to arise predominantly from H atom mobility; all other atom types being ignored.

Supplementary Note 8: Simulated Effect of H/D Exchange in Weakly Hydrated Apoferritin

Because of the preparation protocol of samples (Section Supplementary Methods 4), some H to D exchange of protein hydrogen atoms could occur in the hydrated proteins, involving only solvent-exposed fast exchanging moieties[29].

For apoferritin, considering its labile proton composition, rapidly exchanging hydrogens contribute ~ 10 % of all protein hydrogen atoms. Therefore, at most, only <10% of all protein protons might be expected to exchange. Consequently, for a hydration level of *h*=0.31, to achieve this upper limit, 25% of all solvent deuterium atoms would be required to exchange from D to H. In these conditions the spectral contribution to the scattering spectra may include, at worst, protein proton dynamics, with a maximum H to D protein exchange of 10%, and a few HDO and/or H2O molecules in the hydration shell, with a maximum D to H water exchange of 25%.
To demonstrate, in silico, that such H/D compositional variation has negligible effect on the overall dynamical response (as detected via analysis of the elastic component of the DISF) we computed the MSD(T) at T=290 and 150 K from Protocol 2 simulations of apo_h031, by excluding all fast exchangeable H atoms of apoferritin, the other protein H atoms being included, and including a representative number of H atoms in the solvation shell. The discrepancies of the resulting MSD(T) values compared to those obtained considering only all protein H atoms (and a 100% D_20 solvent layer) are in the most extreme case lower than 10 % (Supplementary Table 4).

T (K)	MSD apo_h031 exchange (Å^2)	MSD apo_h031 no exchange (Å^2)
290	0.463 ± 0.001	0.428 ± 0.001
150	0.124 ± 0.001	0.112 ± 0.001

Supplementary Table 4. In silico MSD(T) values calculated from simulated DISFs for weakly hydrated apoferritin (h=0.31). Data in the first column is obtained by calculating the MSD(T) amplitude of hydrogen atoms in a system which excludes any apoferritin rapidly exchanging labile protons but includes an upper, representative fraction of HDO molecules in the solvation shell which might be expected to arise from the exchange process. Data in the second column considers all protein hydrogen atoms; i.e. no solvent contribution.

Supplementary References:

1 Oleinikova, A., Smolin, N., Brovchenko, I., Geiger, A. & Winter, R. Formation of Spanning Water Networks on Protein Surfaces via 2D Percolation Transition. *The Journal of Physical Chemistry B* **109**, 1988-1998, doi:10.1021/jp045903j (2005).

2 Roh, J. H. *et al.* Onsets of anharmonicity in protein dynamics. *Physical Review Letters* **95**, 038101 (2005).

3 Hong, L., Cheng, X., Glass, D. C. & Smith, J. C. Surface Hydration Amplifies Single-Well Protein Atom Diffusion Propagating into the Macromolecular Core. *Physical Review Letters* **108**, 238102, doi:10.1103/PhysRevLett.108.238102 (2012).

4 Lerbret, A. *et al.* How Strongly Does Trehalose Interact with Lysozyme in the Solid State? Insights from Molecular Dynamics Simulation and Inelastic Neutron Scattering. *The Journal of Physical Chemistry B* **116**, 11103-11116, doi:10.1021/jp3058096 (2012).

5 Hong, L. *et al.* Elastic and Conformational Softness of a Globular Protein. *Physical Review Letters* **110**, 028104, doi:10.1103/PhysRevLett.110.028104 (2013).

6 Fichou, Y., Heyden, M., Zaccai, G., Weik, M. & Tobias, D. J. Molecular Dynamics Simulations of a Powder Model of the Intrinsically Disordered Protein Tau. *The Journal of Physical Chemistry B* **119**, 12580-12589, doi:10.1021/acs.jpcb.5b05849 (2015).

7 de Val, N., Declercq, J.-P., Lim, C. K. & Crichton, R. R. Structural analysis of haemin demetallation by L-chain apoferritins. *Journal of Inorganic Biochemistry* **112**, 77-84, doi:https://doi.org/10.1016/j.jinorgbio.2012.02.031 (2012).

8 Laghaei, R., Evans, D. G. & Coalson, R. D. Metal binding sites of human H-chain ferritin and iron transport mechanism to the ferroxidase sites: a molecular dynamics simulation study. *Proteins* **81**, 1042-1050, doi:10.1002/prot.24251 (2013).

9 Pekar, A. H. & Frank, B. H. Conformation of proinsulin. Comparison of insulin and proinsulin self-association at neutral pH. *Biochemistry* **11**, 4013-4016, doi:10.1021/bi00772a001 (1972).

10 Gursky, O., Badger, J., Li, Y. & Caspar, D. L. Conformational changes in cubic insulin crystals in the pH range 7-11. *Biophys J* **63**, 1210-1220, doi:10.1016/s0006-3495(92)81697-1 (1992).

11 Haas, J. *et al.* Primary Steps of pH-Dependent Insulin Aggregation Kinetics are Governed by Conformational Flexibility. *ChemBioChem* **10**, 1816-1822, doi:https://doi.org/10.1002/cbic.200900266 (2009).

12 Abraham, M. J. *et al.* GROMACS: High performance molecular simulations through multi-level parallelism from laptops to supercomputers. *SoftwareX* **1-2**, 19-25, doi:https://doi.org/10.1016/j.softx.2015.06.001 (2015).

13 Jorgensen, W. L., Maxwell, D. S. & Tirado-Rives, J. Development and Testing of the OPLS All-Atom Force Field on Conformational Energetics and Properties of Organic Liquids. *Journal of the American Chemical Society* **118**, 11225-11236, doi:10.1021/ja9621760 (1996).

14 Jorgensen, W. L., Chandrasekhar, J., Madura, J. D., Impey, R. W. & Klein, M. L. Comparison of simple potential functions for simulating liquid water. *The Journal of Chemical Physics* **79**, 926-935, doi:10.1063/1.445869 (1983).

15 Hess, B., Bekker, H., Berendsen, H. J. C. & Fraaije, J. G. E. M. LINCS: A linear constraint solver for molecular simulations. *Journal of Computational Chemistry* **18**, 1463-1472, doi:https://doi.org/10.1002/(SICI)1096-987X(199709)18:12<1463::AID-JCC4>3.0.CO;2-H (1997).
16 Hockney, R. W. Potential Calculation and some Applications. *Mehods Comput. Phys.* **9**, 135-211 (1970).
17 Essmann, U. *et al.* A smooth particle mesh Ewald method. *The Journal of Chemical Physics* **103**, 8577-8593, doi:10.1063/1.470117 (1995).
18 Bussi, G., Donadio, D. & Parrinello, M. Canonical sampling through velocity rescaling. *The Journal of Chemical Physics* **126**, 014101, doi:10.1063/1.2408420 (2007).
19 Berendsen, H. J. C., Postma, J. P. M., van Gunsteren, W. F., DiNola, A. & Haak, J. R. Molecular dynamics with coupling to an external bath. *The Journal of Chemical Physics* **81**, 3684-3690, doi:10.1063/1.448118 (1984).
20 Humphrey, W., Dalke, A. & Schulten, K. VMD: visual molecular dynamics. *J Mol Graph* **14**, 33-38, 27-38, doi:10.1016/0263-7855(96)00018-5 (1996).
21 Sehnal, D. *et al.* Mol* Viewer: modern web app for 3D visualization and analysis of large biomolecular structures. *Nucleic Acids Res* **49**, W431-w437, doi:10.1093/nar/gkab314 (2021).
22 Kabsch, W. & Sander, C. Dictionary of protein secondary structure: pattern recognition of hydrogen-bonded and geometrical features. *Biopolymers* **22**, 2577-2637, doi:10.1002/bip.360221211 (1983).
23 Goret, G., Aoun, B. & Pellegrini, E. MDANSE: An Interactive Analysis Environment for Molecular Dynamics Simulations. *Journal of Chemical Information and Modeling* **57**, 1-5, doi:10.1021/acs.jcim.6b00571 (2017).
24 Hong, L., Smolin, N., Lindner, B., Sokolov, A. P. & Smith, J. C. Three Classes of Motion in the Dynamic Neutron-Scattering Susceptibility of a Globular Protein. *Physical Review Letters* **107**, 148102, doi:10.1103/PhysRevLett.107.148102 (2011).
25 Telling, M. T. F. & Andersen, K. H. Spectroscopic characteristics of the OSIRIS near-backscattering crystal analyser spectrometer on the ISIS pulsed neutron source. *Physical Chemistry Chemical Physics* **7**, 1255-1261 (2005).
26 MacKerell, A. D., Jr. *et al.* All-Atom Empirical Potential for Molecular Modeling and Dynamics Studies of Proteins. *The Journal of Physical Chemistry B* **102**, 3586-3616, doi:10.1021/jp973084f (1998).
27 Prestrelski, S. J., Tedeschi, N., Arakawa, T. & Carpenter, J. F. Dehydration-induced conformational transitions in proteins and their inhibition by stabilizers. *Biophys J* **65**, 661-671, doi:10.1016/s0006-3495(93)81120-2 (1993).
28 Griebenow, K. & Klibanov, A. M. Lyophilization-induced reversible changes in the secondary structure of proteins. *Proceedings of the National Academy of Sciences* **92**, 10969-10976, doi:doi:10.1073/pnas.92.24.10969 (1995).
29 Bizzarri, A., Paciaroni, A., Arcangeli, C. & Cannistraro, S. Low-frequency vibrational modes in proteins: a neutron scattering investigation. *European Biophysics Journal* **30**, 443-449, doi:10.1007/s002490100167 (2001).
