# Supplementary material for: An experimentally representative in-silico protocol for dynamical studies of lyophilised and weakly hydrated amorphous proteins
Source: Commun Chem. 2024 Apr 12;7:83. doi: 10.1038/s42004-024-01167-6 (PMC11014950; doi:10.1038/s42004-024-01167-6)
Supplement: Supplementary file 2 — Description of Additional Supplementary Files [file 42004_2024_1167_MOESM2_ESM.pdf]

Description of Additional Supplementary Files

File name: Supplementary Data 1

Description: Numerical source for data of figures 1-10

File name: Supplementary Code 1

Description: Supplementary Information Software Python code

MDANSE Python Script
